# 24-Epibrassinolide Enhances Pb-As Phytostabilization in Willow-Vetiver Intercropping Systems: Plant Accumulation Patterns and Soil Biogeochemical Perspectives

**DOI:** 10.3390/plants15213281

**Published:** 2026-10-24

**Authors:** Yepu Li, Lianfu Liang, Qian Dong, Jialu Zhu, Shibo Wei, Mengyuan Chen, Hui Han

**Affiliations:** College of Water Resources and Modern Agriculture, Nanyang Normal University, Nanyang 473061, China; lianglianfu98@163.com (L.L.); 15909543211@163.com (Q.D.); 17395130325@163.com (J.Z.); 13525383102@163.com (S.W.); 19552372295@163.com (M.C.); hanhui2018@nynu.edu.cn (H.H.)

**Keywords:** 24-epibrassinolide, arsenic, lead, phytostabilization, willow–vetiver intercropping

## Abstract

Soil co-contamination with lead (Pb) and arsenic (As) poses severe environmental risks. This study investigated the effects of 24-epibrassinolide (24-Brs) on Pb and As accumulation in willow (*Salix babylonica* × *S.* × *leucopithecia* ‘NJU513’) and vetiver grass (*Chrysopogon zizanioides*) under monoculture and intercropping in naturally contaminated soil (215.48 mg Pb kg^−1^, 120.16 mg As kg^−1^). Plant biomass, metal accumulation, soil physicochemical properties, metal fractionation, and mineral nutrient dynamics were evaluated. Exogenous 24-Brs significantly promoted plant biomass under Pb-As stress (*p* < 0.05), with the greatest increase up to 114.81% in vetiver monoculture (vs. untreated stress control, NBrs). 24-Brs increased root Pb concentrations in willow (14.23 to 24.55 mg kg^−1^) and intercropped vetiver (44.51 to 68.58 mg kg^−1^), and root As in willow (10.80 to 13.55 mg kg^−1^, not significant). Soil fractionation revealed that 24-Brs shifted Pb and As toward more bioavailable fractions, with exchangeable Pb increasing by 63.22% in willow-planted soil. 24-Brs also reshaped essential mineral nutrient (Zn, Cu, Ni, Mn) fractionation and uptake patterns. Intercropping combined with 24-Brs achieved the lowest As leaching (1.50 μg L^−1^, a 90.20% reduction compared with the unplanted control at 15.31 μg L^−1^), although the difference between 24-Brs and NBrs treatments within each planting pattern was not statistically significant. These findings indicate that 24-Brs enhances Pb–As phytostabilization through combined effects on plant metal accumulation and soil biogeochemistry, although the underlying plant physiological mechanisms require further direct investigation.

## 1. Introduction

Soil contamination with lead (Pb) and arsenic (As) represents one of the most intractable environmental challenges of our time. Mining operations, smelting activities, pesticide application, and industrial emissions have left vast expanses of land co-contaminated with Pb and As, particularly in developing economies where regulatory systems have historically lagged behind industrial expansion [1]. The coexistence of these two toxic elements is especially concerning because Pb and As exhibit distinctly different geochemical behaviors. Pb tends to exist as a cationic species and is relatively immobilized under oxidizing conditions, whereas As occurs as anionic oxyanions whose mobility is enhanced under reducing environments, rendering the remediation of co-contamination far more complex than that of single-element pollution [2]. Conventional engineering approaches such as soil washing, vitrification, and excavation are costly at large scales and often disrupt soil structure and biological function [3]. In contrast, phytoremediation offers a solar-driven, eco-friendly alternative that can be applied in situ with minimal disturbance to soil ecosystems [4].

Among plant combinations used for remediating metal-contaminated soils, willow (*Salix* spp.) and vetiver grass (*Chrysopogon zizanioides*) stand out for their complementary traits, making their combination particularly well suited for Pb-As co-contaminated sites. Willow is a fast-growing woody plant with deep roots and high transpiration rates, and has been shown to accumulate multiple trace elements in aboveground biomass, including Pb, cadmium (Cd), zinc (Zn), and copper (Cu) [5]. Several willow clones have been field-validated for phytoremediation of industrial brownfields and tailings, achieving Pb removal rates of up to 63% over multiple annual rotations [6]. Vetiver grass, native to South Asia, is a perennial C_4_ plant with a dense, extensive root system that can penetrate 3–5 m into the soil profile and tolerates an exceptionally wide range of soil stresses, including extreme pH, high salinity, and heavy metals (Pb) [7]. Critically, the tolerance thresholds of vetiver far exceed typical regulatory investigation levels for As (estimated toxicity thresholds between 100 and 250 mg kg^−1^) and other metals, making it suitable for sites with moderate to heavy contamination loads [8]. Vetiver grass primarily retains metals in root tissues, making it well suited for phytostabilization strategies aimed at preventing contaminant migration rather than removal through harvesting.

Tree–herb intercropping systems exploit multiple synergistic effects that are particularly advantageous for the remediation of Pb-As co-contamination. First, the two species occupy complementary rooting zones. Willow develops a woody taproot system that penetrates deep into the soil, whereas vetiver grass forms a dense fibrous root mat in the upper soil layers, achieving three-dimensional soil coverage that maximizes metal interception and rhizosphere volume. This spatial complementarity is especially valuable for As contamination, where preventing vertical migration to groundwater is a primary remediation target. Second, the two species employ different metal accumulation strategies: willow exhibits moderate phytoextraction for more mobile metals and phytostabilization for Pb and As [5], whereas vetiver grass is a classic phytostabilization species that retains most contaminants within its extensive root network. This dual-strategy system simultaneously provides contaminant removal (through harvesting willow biomass, albeit limited for Pb and As) and immobilization (through vetiver root retention), thereby achieving both purification and risk reduction goals. Third, the willow–vetiver combination offers ecological robustness and practical scalability. Both species are easily propagated (willow through cuttings, vetiver through seedlings) and have demonstrated field feasibility across diverse climatic and soil conditions [9]. The woody component (willow) provides long-term site structure and harvestable biomass through coppicing, whereas the herbaceous component (vetiver) provides rapid soil cover, erosion control, and immediate stabilization. This combination is particularly applicable to tailings, industrial brownfields, and contaminated riparian zones where both erosion control and metal immobilization are needed. However, the specific interactions between willow and vetiver grass under Pb-As co-contamination, and the regulatory potential of brassinosteroids on this system, remain largely unexplored. For clarity, phytostabilization refers to the immobilization of contaminants primarily in plant roots and the rhizosphere, with minimal aboveground accumulation and no removal through harvesting; phytoextraction, by contrast, involves the uptake and translocation of contaminants into aboveground tissues followed by harvest removal. The willow–vetiver combination used here is therefore best characterized as a phytostabilization-oriented system [3,5,9].

In recent years, the combination of woody and herbaceous species in intercropping systems has gained attention as a strategy to maximize phytoremediation efficiency while improving soil health and structural stability. Tree–herb intercropping can increase total metal extraction through complementary rooting depths, rhizosphere effects that alter metal bioavailability, and enhanced rhizosphere microbial activity [10]. A recent study by Zhang et al. [11] showed that intercropping willow with herbaceous plants such as ryegrass (*Lolium perenne* L.) and beggarticks (*Bidens pilosa*) significantly increased total Cd and Pb removal compared with monocultures, driven in part by root exudate-mediated changes in metal speciation. However, intercropping effects are highly context-dependent, and the specific interactions between willow and vetiver grass under Pb-As co-contamination remain largely uncharacterized. Even with appropriate plant species selection, phytoremediation efficiency is often constrained by two major bottlenecks: phytotoxicity at high metal concentrations and limited metal bioavailability in the soil matrix [12]. A growing body of research indicates that the exogenous application of plant growth regulators can simultaneously address both constraints [13]. For example, Emamverdian et al. (2022) demonstrated that benzylaminopurine and abscisic acid could mitigate Cd and Cu toxicity in Pleioblastus pygmaeus by improving growth performance and antioxidant capacity while modifying metal accumulation and translocation patterns [14]. More recently, Emamverdian et al. (2024) provided direct evidence that jasmonates and brassinosteroids alleviate Pb toxicity in bamboo through enhanced antioxidant defense and modified Pb translocation [15]. Brassinosteroids (BRs) are a class of polyhydroxylated steroid hormones widely distributed in the plant kingdom that have attracted considerable attention for their multifaceted roles in plant growth regulation and stress adaptation [16]. 24-Epibrassinolide (24-Brs), one of the most biologically active BRs, has been shown to alleviate heavy metal toxicity through multiple mechanisms, including the enhancement of antioxidant defense systems, regulation of metal chelation pathways, and modulation of nutrient uptake [17]. A meta-analysis encompassing 73 studies confirmed that under heavy metal stress, 24-Brs application increased shoot and root length by an average of 47.90% and reduced metal concentrations in plant tissues by 32.90%, although the effects varied by metal type and plant family [18]. Whereas the protective effects of 24-Brs against metal stress in crop plants are well documented, its potential to enhance phytoremediation in contaminated soils is only beginning to be explored. Several recent studies suggest that 24-Brs may paradoxically increase metal accumulation in certain hyperaccumulator or phytostabilization species while simultaneously improving stress tolerance, a combination highly desirable for remediation purposes [19]. Yang et al. [20] reported that the combined application of 24-Brs with phosphate-solubilizing bacteria increased As accumulation in *Sedum lineare* by 97.30% compared with the control, highlighting the hormone’s ability to both mobilize soil As and promote plant uptake. However, existing studies of 24-Brs in phytoremediation have largely focused on single-metal systems, and the influence of planting pattern and species interactions on 24-Brs efficacy remains poorly understood [21], and information on Pb-As co-contamination is markedly scarce. Equally important but even less studied are the cascading effects of 24-Brs on soil metal speciation and mineral nutrient dynamics. Heavy metals interact with essential mineral elements at multiple levels: they compete for transporters, disrupt enzyme function, and alter soil biogeochemical processes that regulate nutrient availability [22]. BRs are known to regulate the expression of genes encoding nutrient transporters and to influence root architecture in ways that may reshape the mineral economy of plant–soil systems [23]. To our knowledge, however, no comprehensive study has simultaneously tracked how 24-Brs affects the speciation of both contaminant metals (Pb, As) and essential nutrients (Zn, Cu, Ni, Mn, Ca) in soil, and how these speciation changes translate into altered plant uptake under intercropping conditions.

In addition, although these studies have demonstrated the efficacy of 24-Brs in enhancing phytoremediation of Cd-contaminated soils through willow–alfalfa intercropping systems [24,25], several critical knowledge gaps remain. First, the applicability of 24-Brs to other plant combinations, particularly those involving deep-rooted woody species paired with dense fibrous-rooted grasses, has not been explored. Second, no study to date has investigated whether 24-Brs can enhance phytoremediation efficiency under Pb–As co-contamination, where the interactions between two toxic elements with contrasting chemical behaviors (cationic Pb vs. anionic As) may fundamentally alter plant uptake dynamics and soil biogeochemical processes. Third, the potential of 24-Brs to mitigate metal(loid) leaching losses in intercropping systems remains entirely unknown. To address these gaps, we hypothesized that combining 24-Brs application with a willow–vetiver intercropping system could enhance Pb and As phytostabilization while simultaneously reducing leaching risks in co-contaminated soils.

Building on these knowledge gaps, a rhizosphere-focused pot experiment was conducted to evaluate the combined effects of 24-Brs application and planting patterns (willow monoculture, vetiver monoculture, and willow–vetiver intercropping) on naturally Pb-As co-contaminated soil. The following hypotheses were tested: (i) 24-Brs would alleviate Pb-As phytotoxicity and increase the biomass of both species; (ii) 24-Brs would shift soil Pb and As toward more bioavailable fractions, thereby enhancing plant metal accumulation; (iii) intercropping would produce complementary remediation effects relative to monocultures; and (iv) 24-Brs would differentially regulate the speciation and uptake of essential mineral nutrients, with important implications for plant nutrition under metal stress. To test these hypotheses, soil physicochemical parameters, Tessier sequential extraction fractions of Pb, As, and four mineral elements, plant biomass, metal concentrations in different plant tissues, and Pb and As concentrations in soil leachate were determined.

## 2. Results

### 2.1. Soil Physicochemical Properties

**Figure 1:**
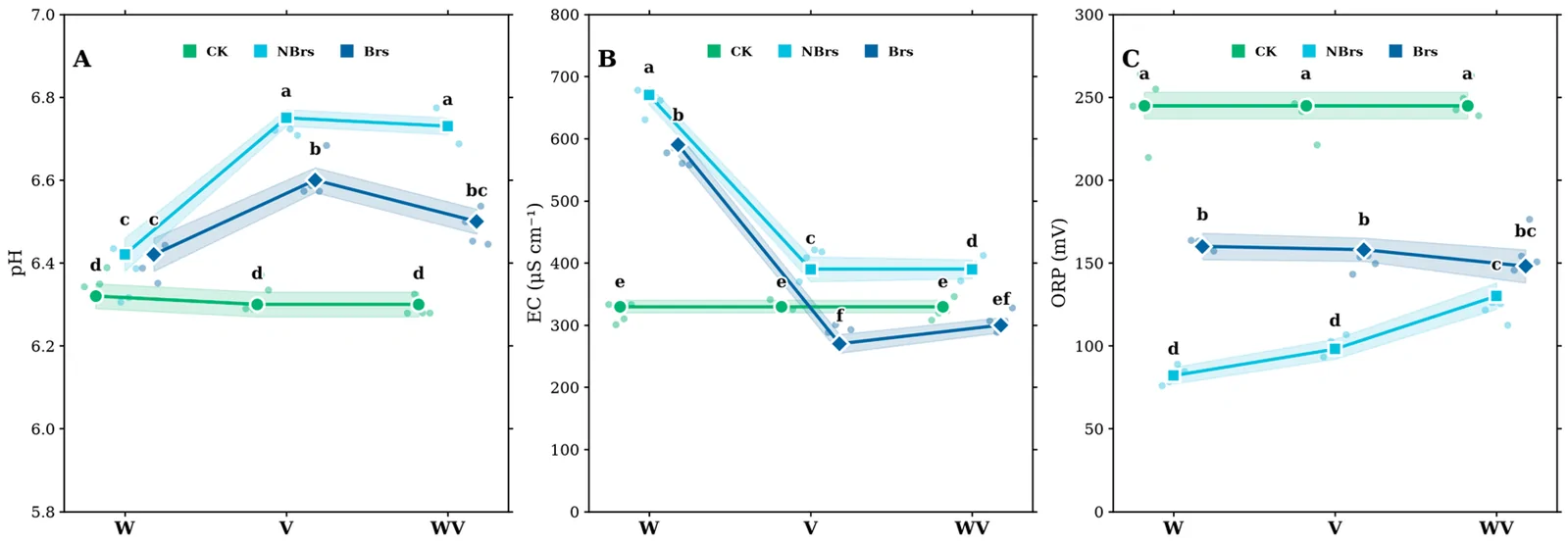
Effects of 24-epibrassinolide application on soil pH (**A**), electrical conductivity (EC, (**B**)), and oxidation–reduction potential (ORP, (**C**)). W, V, and WV indicate that willow, vetiver, and willow-vetiver are planted in the soil, respectively. CK indicates the polluted soil without plants. NBrs and Brs indicate that plants are treated without and with 24-epibrassinolide, respectively. The different letters indicate the significant differences at *p* < 0.05 levels among different treatments.

Plants and 24-Brs significantly altered soil pH and EC, and 24-Brs significantly increased soil ORP, although the effects on pH and EC varied by planting pattern (Figure 1).

The unplanted control had a pH of 6.31. Plants elevated the pH, most notably in systems containing vetiver grass (V-NBrs: 6.75; WV-NBrs: 6.74). 24-Brs significantly decreased the pH in vetiver monoculture (6.60; −2.22%; *p* < 0.05) and intercropping (6.51; −3.41%; *p* < 0.05), but had no significant effect on willow monoculture. EC increased under Pb-As contamination, whereas 24-Brs significantly decreased EC across all planting patterns (*p* < 0.05), with the largest reduction observed in V-Brs (265 vs. 395 μS cm^−1^; −32.91%). Planting reduced the ORP from 244 mV (CK) to 82–130 mV under NBrs. 24-Brs significantly increased the ORP in all patterns (*p* < 0.05): relative to NBrs, W-Brs reached 160 mV (+95.12%), V-Brs reached 157 mV (+60.20%), and WV-Brs reached 148 mV (+13.85%).

### 2.2. Plant Biomass Responses

**Figure 2:**
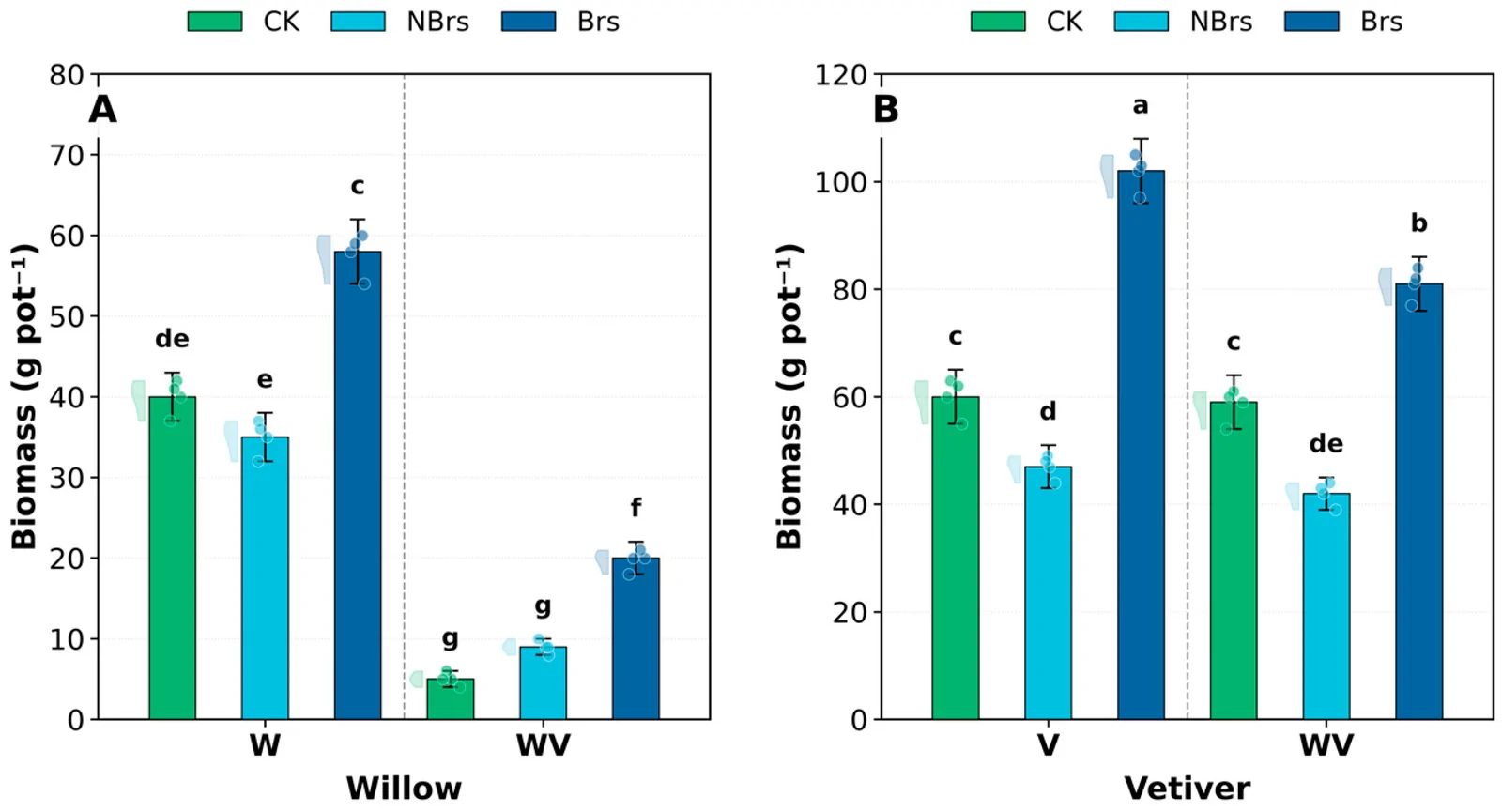
Effects of 24-epibrassinolide application on willow (**A**) and vrtiver (**B**) total biomass. W, V, and WV indicate that willow, vetiver, and willow-vetiver are planted in the soil, respectively. CK indicates the unpolluted soil with plants. NBrs and Brs indicate that plants are treated without and with 24-epibrassinolide, respectively. The different letters indicate the significant differences at *p* < 0.05 levels among different treatments.

24-Brs strongly alleviated Pb-As-induced growth inhibition in both species and both planting patterns (Figure 2).

Under monoculture conditions, Pb-As stress reduced willow biomass by 12.44% (not significant) and vetiver grass biomass by 21.31% (*p* < 0.05). 24-Brs substantially increased biomass relative to NBrs: willow monoculture +63.89% (59.33 vs. 36.12 g pot^−1^; *p* < 0.05), vetiver monoculture +114.81% (103.56 vs. 48.21 g pot^−1^; *p* < 0.05). Under intercropping, willow biomass was greatly reduced due to asymmetric competition, but 24-Brs still doubled it (+110.53%; *p* < 0.05), whereas intercropped vetiver biomass increased by 93.71% (81.90 vs. 42.28 g pot^−1^; *p* < 0.05). The total system biomass under 24-Brs was highest in vetiver monoculture (103.56 g pot^−1^, V-Brs), which exceeded all other planting systems.

### 2.3. Lead and Arsenic Accumulation in Willow

**Figure 3:**
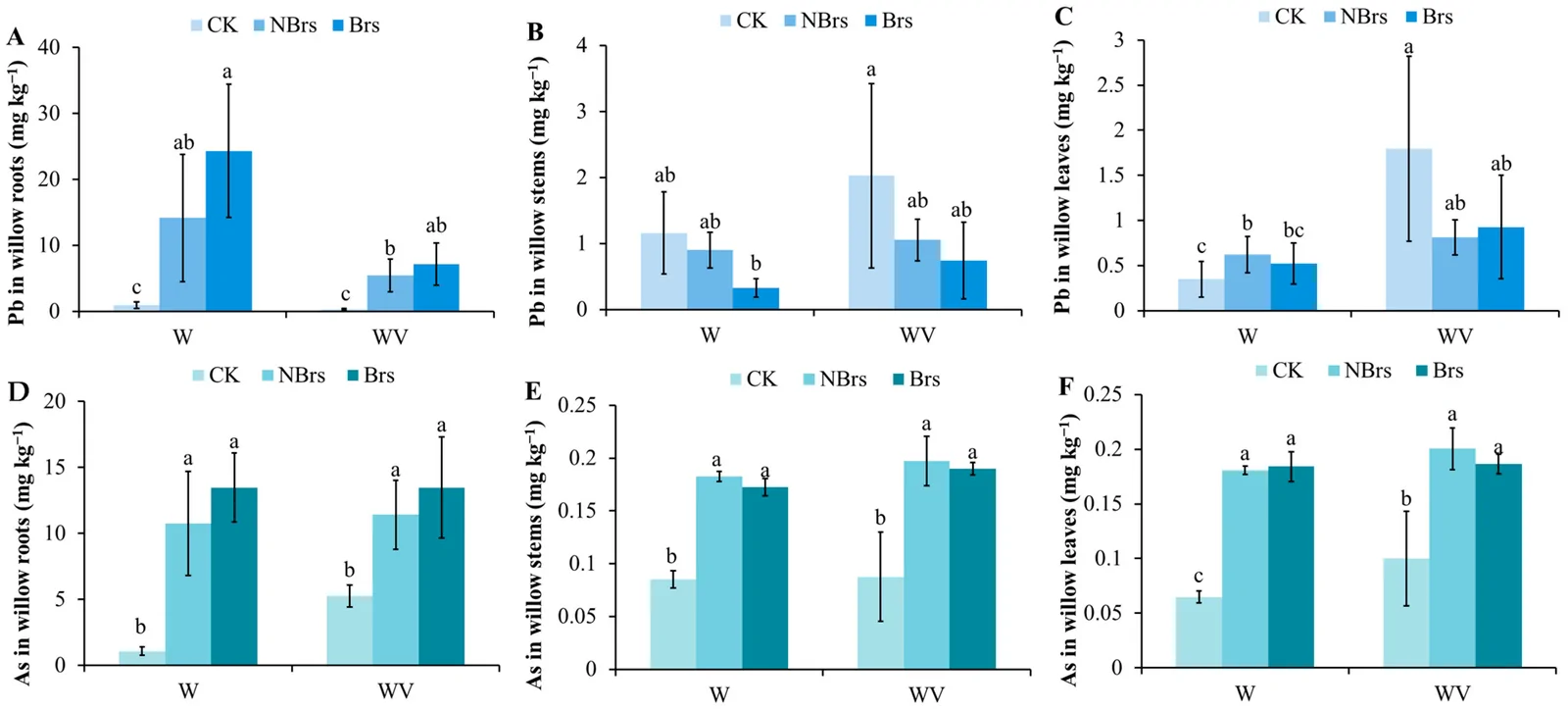
Effects of 24-epibrassinolide application on Pb (**A**–**C**) and As (**D**–**F**) concentrations in willow roots, stems and leaves. W and WV indicate that willow and willow-vetiver are planted in the soil, respectively. CK indicates the unpolluted soil with plants. NBrs and Brs indicate that plants are treated without and with 24-epibrassinolide, respectively. The different letters indicate the significant differences at *p* < 0.05 levels among different treatments.

24-Brs increased root Pb and As concentrations in willow while reducing Pb translocation to aboveground tissues (Figure 3).

Willow roots accumulated the highest Pb concentrations. Under monoculture, 24-Brs increased root Pb from 14.23 to 24.55 mg kg^−1^ (+72.54%), although this increase did not reach statistical significance (*p* = 0.096), whereas stem Pb decreased from 0.90 to 0.37 mg kg^−1^ (−58.89%), indicating enhanced phytostabilization. Under intercropping, root Pb was generally lower (NBrs: 5.53; Brs: 7.24 mg kg^−1^), and leaf Pb was the lowest among all tissues.

As was predominantly retained in willow roots (10.83–13.61 mg kg^−1^ under contaminated conditions), approximately one order of magnitude higher than in the uncontaminated control. 24-Brs increased root As in both planting patterns (to 13.55 and 13.57 mg kg^−1^ in monoculture and intercropping, respectively), although not significantly. Stem and leaf As remained at very low levels under contaminated conditions (0.17–0.20 mg kg^−1^).

### 2.4. Lead and Arsenic Accumulation in Vetiver Grass

**Figure 4:**
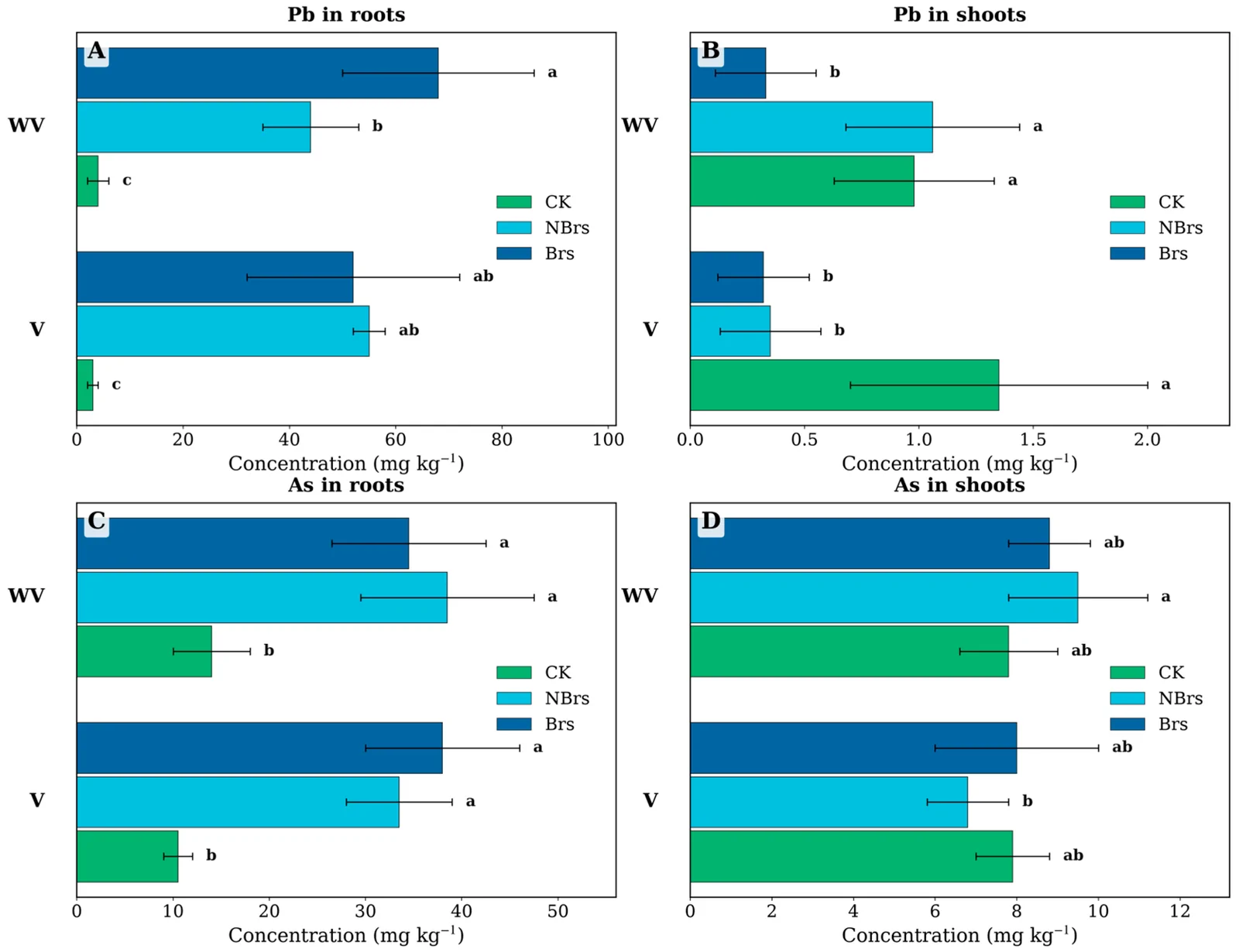
Effects of 24-epibrassinolide application on Pb (**A**,**B**) and As (**C**,**D**) concentrations in vetiver roots and shoots. V and WV indicate that vetiver and willow–vetiver are planted in the soil, respectively. CK indicates the unpolluted soil with plants. NBrs and Brs indicate that plants are treated without and with 24-epibrassinolide, respectively. The different letters indicate the significant differences at *p* < 0.05 levels among different treatments.

Vetiver grass primarily accumulated Pb and As in roots, and 24-Brs further increased root Pb under intercropping (Figure 4).

Root Pb reached 55.56 mg kg^−1^ under vetiver monoculture NBrs treatment, and 44.51 mg kg^−1^ under intercropping NBrs. 24-Brs increased root Pb to 68.58 mg kg^−1^ under intercropping (+54.08%), although this increase did not reach statistical significance (*p* = 0.076 for the interaction), while slightly decreasing it in monoculture (53.49 mg kg^−1^). Shoot Pb was very low in all contaminated treatments (0.32–0.55 mg kg^−1^), and 24-Brs further reduced it.

Root As in vetiver grass under contaminated conditions was 33.83–42.81 mg kg^−1^, approximately 3–4 times that of willow roots. 24-Brs slightly increased root As in monoculture (to 38.26 mg kg^−1^) but not under intercropping. Shoot As in contaminated soil was 6.87–9.47 mg kg^−1^, and 24-Brs caused a slight reduction.

### 2.5. Soil Pb and As Fractionation

**Figure 5:**
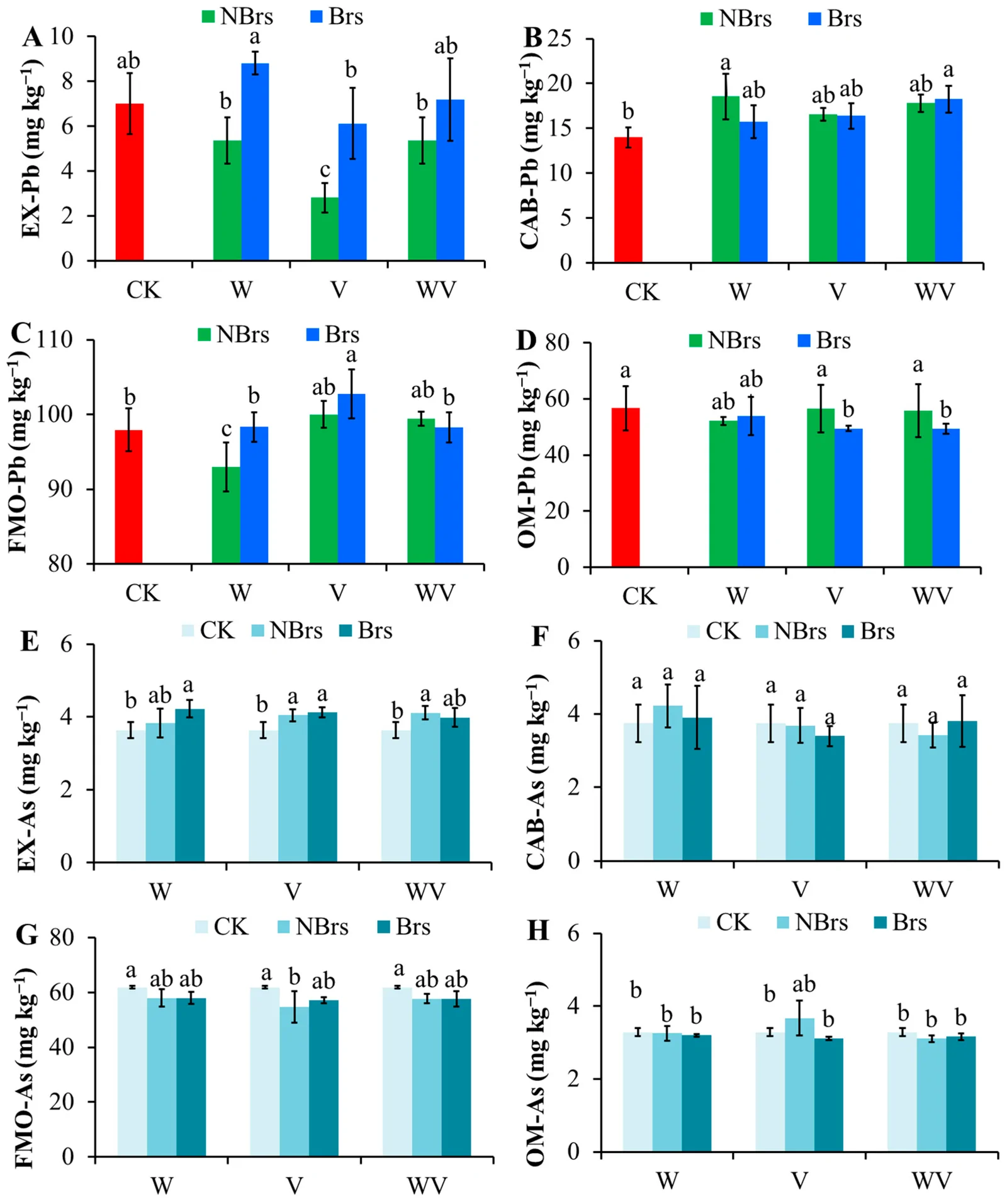
Effects of 24-epibrassinolide application on the concentrations of soil EX-Pb/As (**A**,**E**), CAB-Pb/As (**B**,**F**), FMO-Pb/As (**C**,**G**), and OM-Pb/As (**D**,**H**). W, V, and WV indicate that willow, vetiver, and willow–vetiver are planted in the soil, respectively. CK indicates the polluted soil without plants. NBrs and Brs indicate that plants are treated without and with 24-epibrassinolide, respectively. The red color in figure (**A**–**D**) indicate CK treatment. The different letters indicate the significant differences at *p* < 0.05 levels among different treatments.

24-Brs substantially reshaped the geochemical fractions of Pb and As, shifting both elements toward more bioavailable fractions (Figure 5).

In unplanted soil, Pb was mainly distributed in the FMO and OM fractions, with EX-Pb accounting for about 3.25% of the total. 24-Brs significantly increased EX-Pb across all planting patterns (*p* < 0.05): willow soil +63.22% (8.83 vs. 5.41 mg kg^−1^), vetiver soil +118.37% (6.18 vs. 2.83 mg kg^−1^), intercropping +33.33% (7.24 vs. 5.43 mg kg^−1^). OM-Pb correspondingly decreased, indicating that 24-Brs primarily mobilized Pb from the organic-bound pool. As fractions were dominated by FMO (accounting for approximately 43–52% of the total), with EX-As representing only 3.05–4.23%. 24-Brs significantly increased EX-As in willow soil while partially restoring FMO-As concentrations. CAB-As and OM-As showed little variation among treatments.

### 2.6. Mineral Nutrient Concentrations in Plants and Soil

**Figure 6:**
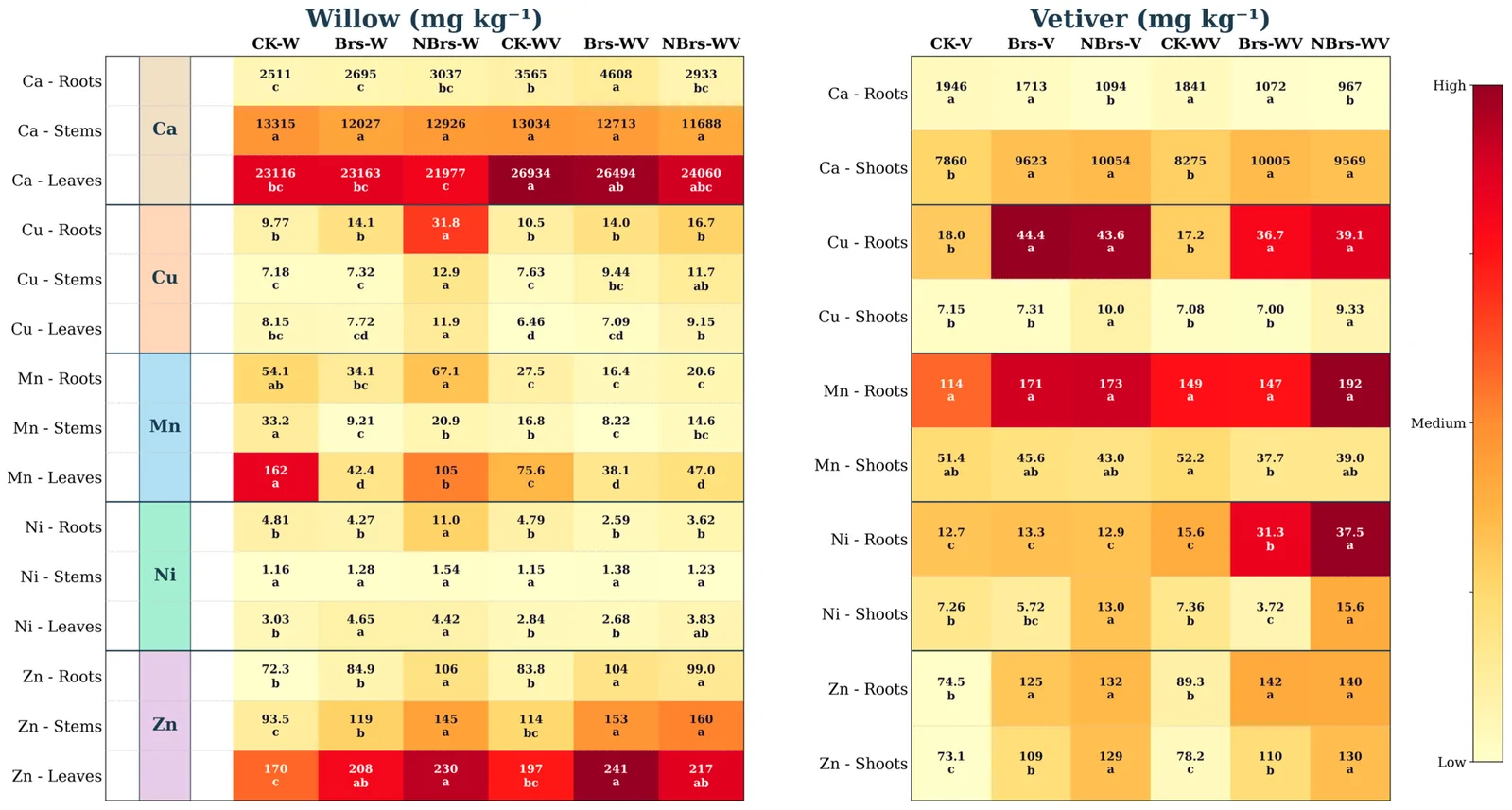
Effects of 24-epibrassinolide application on the concentrations of Ca, Cu, Mn, Ni, and Zn in willow and vetiver tissues. W, V, and WV indicate that willow, vetiver, and willow-vetiver are planted in the soil, respectively. CK indicates the unpolluted soil with plants. NBrs and Brs indicate that plants are treated without and with 24-epibrassinolide, respectively. The different letters indicate the significant differences at *p* < 0.05 levels among different treatments.

**Figure 7:**
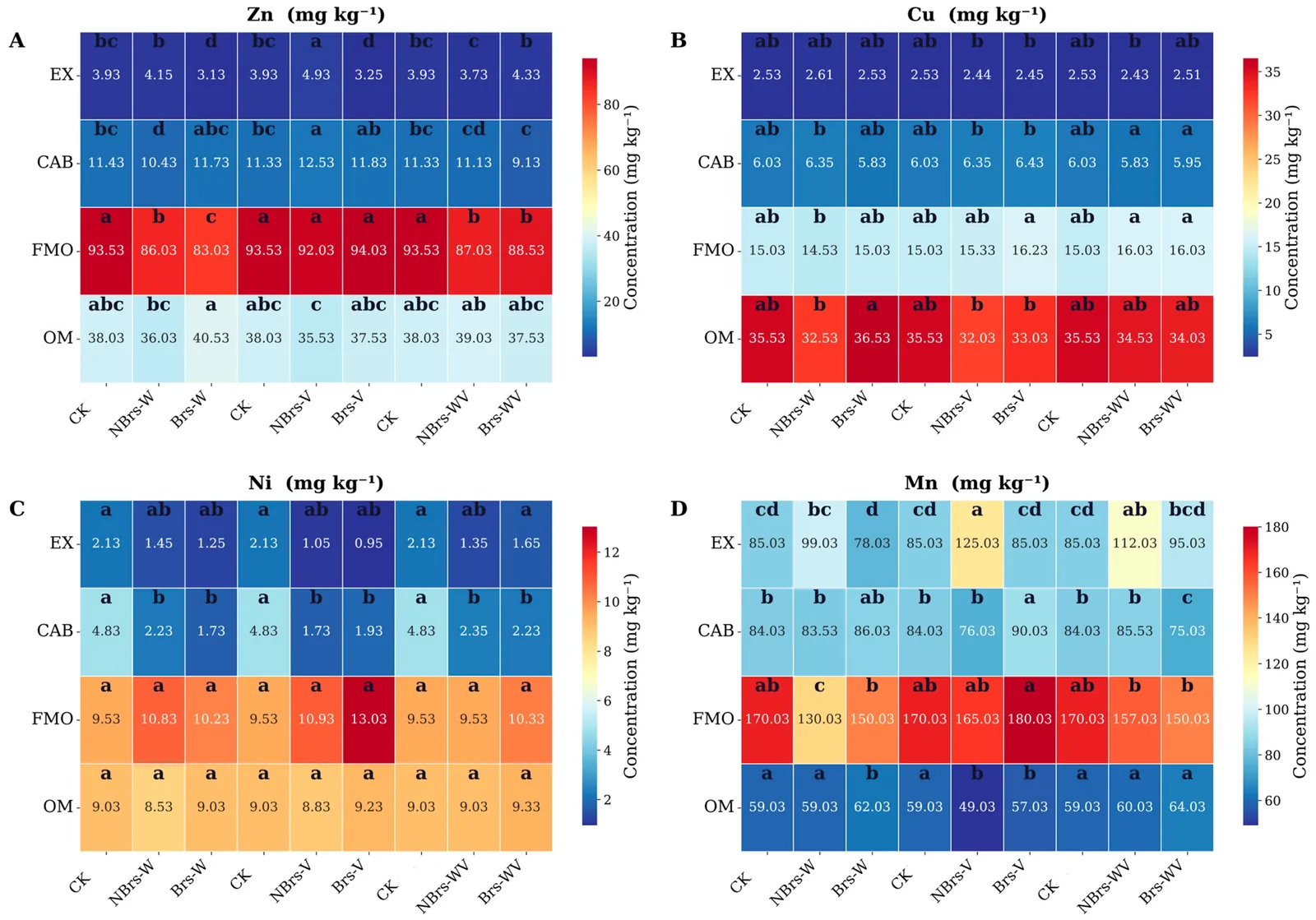
Effects of 24-epibrassinolide application on the concentrations of soil EX-Zn/Cu/Ni/Mn, CAB-Zn/Cu/Ni/Mn, FMO-Zn/Cu/Ni/Mn, and OM-Zn/Cu/Ni/Mn. W, V, and WV indicate that willow, vetiver, and willow-vetiver are planted in the soil, respectively. (**A**–**D**) indicate soil fractionations of Zn, Cu, Ni, and Mn, respectively. CK indicates the polluted soil without plants. NBrs and Brs indicate that plants are treated without and with 24-epibrassinolide, respectively. The different letters indicate the significant differences at *p* < 0.05 levels among different treatments.

Pb-As contamination and 24-Brs application differentially affected the concentrations of essential mineral nutrients (Zn, Cu, Ni, Mn, Ca) in plant tissues and soil fractions (Figure 6 and Figure 7, Tables S2 and S3). Responses were element-specific, tissue-dependent, and modulated by the planting pattern.

#### 2.6.1. Mineral Nutrient Dynamics in Willow

Calcium was the most abundant mineral element in willow, with the highest concentrations observed in leaves (21,977–26,934 mg kg^−1^). Pb-As contamination did not significantly alter Ca concentrations in stems or leaves, whereas 24-Brs significantly increased root Ca to 4608 mg kg^−1^ under intercropping (*p* < 0.05), enhancing root Ca retention.

Copper was highest in willow roots. Pb-As contamination dramatically increased root Cu from 9.77 to 31.77 mg kg^−1^ in monoculture (+225.18%; *p* < 0.05), whereas 24-Brs reversed it to 14.08 mg kg^−1^ (−55.68%; *p* < 0.05). A similar pattern was observed in stems and leaves, although the responses were more moderate under intercropping.

Manganese had the highest concentrations in willow leaves (38.09–162.44 mg kg^−1^). Pb-As contamination significantly reduced leaf Mn by 35.41%, and 24-Brs further decreased it to 42.40 mg kg^−1^ (only 26.10% of CK; *p* < 0.05). Intercropping itself also substantially reduced willow Mn concentrations (*p* < 0.05).

Nickel was highest in willow roots (2.59–11.02 mg kg^−1^). Pb-As contamination increased root Ni from 4.81 to 11.02 mg kg^−1^ in monoculture (+129.11%; *p* < 0.05), whereas 24-Brs restored it to near CK levels (4.27 mg kg^−1^; *p* < 0.05 vs. NBrs). Ni responses were more moderate under intercropping.

Zinc was highest in willow leaves (170.03–241.37 mg kg^−1^). Pb-As contamination significantly increased Zn in all tissues (*p* < 0.05), and in most cases 24-Brs maintained Zn at intermediate levels between CK and NBrs. 24-Brs also enhanced Zn translocation to stems under both planting patterns.

#### 2.6.2. Mineral Nutrient Dynamics in Vetiver Grass

Calcium was highest in vetiver shoots (7860–10,054 mg kg^−1^), whereas root Ca was substantially lower (967–1946 mg kg^−1^). Pb-As contamination significantly reduced root Ca in monoculture by 43.78% but increased shoot Ca (*p* < 0.05), indicating altered Ca allocation favoring translocation to shoots under stress.

Copper mainly accumulated in vetiver roots. Contamination stress increased root Cu from 17.96 to 43.63 mg kg^−1^ (+142.93%; *p* < 0.05), and 24-Brs did not further alter root Cu. However, 24-Brs significantly reduced shoot Cu to near CK levels under both planting patterns (*p* < 0.05).

Manganese concentrations in vetiver roots (114.07–191.96 mg kg^−1^) were higher than in shoots (37.68–52.25 mg kg^−1^), contrary to the pattern in willow. Neither contamination nor 24-Brs significantly altered root Mn, but 24-Brs significantly reduced shoot Mn under intercropping (*p* < 0.05), consistent with the reduction in soil EX-Mn.

Nickel exhibited the most pronounced intercropping effect: root Ni in vetiver monoculture remained at approximately 12.67–13.29 mg kg^−1^, whereas intercropping increased it to 37.48 mg kg^−1^ (WV-NBrs) and 31.26 mg kg^−1^ (WV-Brs), representing increases of 190% and 135%, respectively (*p* < 0.05). 24-Brs significantly reduced shoot Ni under both planting patterns (*p* < 0.05).

Both root and shoot Zn in vetiver grass increased significantly under Pb-As contamination (*p* < 0.05), with root Zn in monoculture increasing from 74.55 to 132.30 mg kg^−1^. 24-Brs maintained Zn at levels slightly lower than NBrs but still higher than the control, with a significant decrease observed in shoots.

#### 2.6.3. Soil Mineral Nutrient Fractions

Sequential extraction (Tessier) results showed that 24-Brs application significantly reshaped the chemical fractions of Zn, Cu, Ni, and Mn in soil, with the most pronounced changes occurring in the exchangeable (EX) and Fe/Mn oxide-bound (FMO) fractions (Figure 7).

Zn fractions were dominated by FMO and OM components across all treatments. Pb-As contamination significantly increased EX-Zn in both willow and vetiver monocultures (*p* < 0.05), whereas 24-Brs decreased EX-Zn and concomitantly increased FMO-Zn in vetiver and intercropping soils, consistent with redox-mediated Zn immobilization.

Ni fractions responded most strongly to the planting pattern. EX-Ni and CAB-Ni in intercropping soil were significantly higher than in monoculture soils (*p* < 0.05), consistent with the substantial increase in Ni uptake by intercropped vetiver roots. 24-Brs promoted a moderate shift from exchangeable to FMO-Ni in vetiver monoculture, indicating oxidative immobilization.

Mn exhibited the most dramatic fractionation changes. 24-Brs consistently decreased EX-Mn (*p* < 0.05) while increasing FMO-Mn (*p* < 0.05) across all planting patterns; for example, under vetiver monoculture EX-Mn decreased from 125.78 to 85.91 mg kg^−1^, while FMO-Mn increased from 161.47 to 179.82 mg kg^−1^. This shift from EX to FMO is consistent with the 24-Brs-induced increase in soil ORP.

### 2.7. As and Pb Concentrations in Soil Leachate

**Figure 8:**
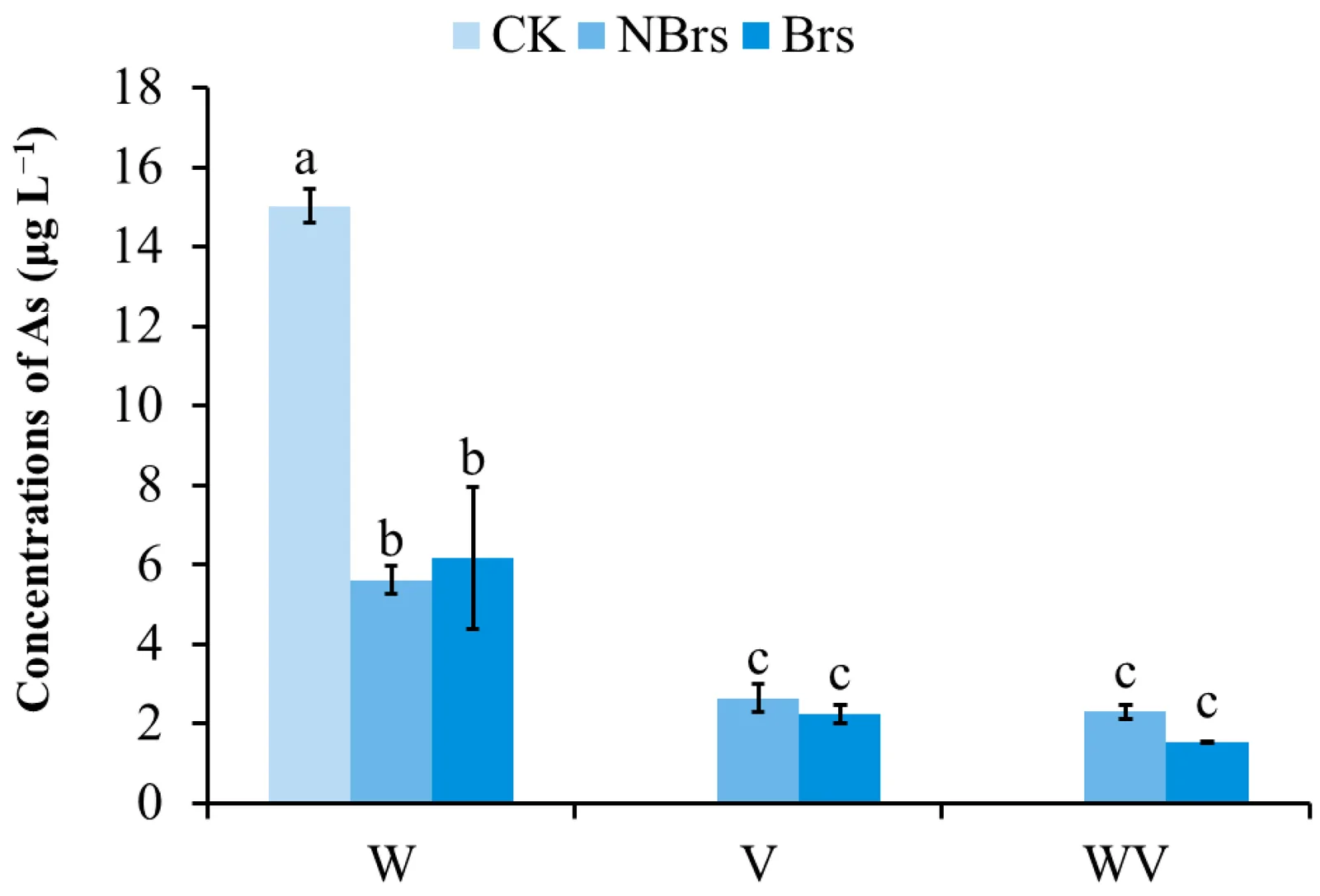
Effects of 24-epibrassinolide application on the concentrations of As in leachate. W, V, and WV indicate that willow, vetiver, and willow–vetiver are planted in the soil, respectively. CK indicates the polluted soil without plants. NBrs and Brs indicate that plants are treated without and with 24-epibrassinolide, respectively. The different letters indicate the significant differences at *p* < 0.05 levels among different treatments.

The intercropping system combined with 24-Brs achieved the lowest As leaching, reducing it by more than 90% compared with the unplanted control (Figure 8). Pb concentrations in all treatments were below the ICP-OES detection limit (LOD = 3.20 μg L^−1^). However, the difference in leachate As between 24-Brs and NBrs treatments within each planting pattern was not statistically significant.

Leachate As was highest in unplanted contaminated soil, at 15.31 μg L^−1^. Plants substantially reduced leaching: willow monoculture reduced it by 63.33% (5.61 μg L^−1^), vetiver monoculture by 82.67% (2.65 μg L^−1^), and intercropping by 84.67% (2.35 μg L^−1^), with vetiver performing significantly better than willow (*p* < 0.05). However, 24-Brs did not significantly affect leachate As concentrations within any planting pattern (*p* > 0.05). In willow monoculture, the Brs treatment showed a slightly higher mean leachate As than NBrs, though the difference was not significant. In vetiver monoculture and intercropping, Brs treatments showed numerically lower leachate As than NBrs, but these differences were also not statistically significant. The intercropping system with 24-Brs (Brs-WV) achieved the lowest absolute leachate As concentration (1.50 μg L^−1^), representing a 90.20% reduction compared with the unplanted control.

## 3. Discussion

Before presenting the discussion, it is important to acknowledge the scope of the present study. No molecular-level analyses (transcriptomics, proteomics, or gene expression assays) were performed. Accordingly, all mechanistic explanations involving antioxidant enzymes, metal transporters, signaling pathways, or transcription factor regulation are presented as working hypotheses inferred from the observed physiological and geochemical patterns, in conjunction with published literature. These hypotheses require direct experimental validation in future studies.

### 3.1. 24-Brs Alleviates Pb-As Phytotoxicity Through Growth Promotion and Enhanced Stress Tolerance

Pb-As co-contamination significantly inhibited plant growth, with vetiver grass being particularly affected under monoculture conditions, showing a 21.31% biomass decline. Exogenous application of 24-Brs significantly reversed this growth inhibition, with increases of 63.89% in willow monoculture and 114.81% in vetiver monoculture relative to their respective NBrs controls (*p* < 0.05). Under intercropping, 24-Brs increased willow biomass by 110.53% and vetiver biomass by 93.71%, demonstrating that the hormone effectively alleviates Pb-As phytotoxicity across different species and planting configurations. This overall phenotypic recovery is supported by two complementary mechanisms: direct growth promotion and enhanced stress tolerance. This finding aligns with the established consensus in the literature on the dual protective role of brassinosteroids under heavy metal stress, promoting growth while enhancing defenses [26].

Brassinosteroids exert well-known effects on cell elongation and division through the BRI1–BSU1–BIN2 signaling cascade, which activates BZR1/BES1 transcription factors regulating genes involved in cell wall loosening, photosynthesis, and biomass accumulation [26,27]. The substantial biomass increase observed in this study (up to 114.81% in vetiver monoculture) is consistent with this classic growth-promoting function of BRs. By maintaining photosynthetic machinery and cell expansion under Pb-As stress, conditions that would otherwise suppress them, 24-Brs provides the metabolic platform required for both growth and stress defense. However, it should be noted that these signaling components were not directly measured in the present study, and the above description serves as a hypothetical framework rather than a demonstrated mechanism.

In addition to growth promotion, 24-Brs may enhance plant tolerance to Pb-As stress through mechanisms that could include enhanced antioxidant defense and osmotic regulation, as reported in other species [28], although these were not directly measured in the present study. This interpretation is consistent with recent reports suggesting that plant growth regulators, including brassinosteroids and jasmonates, may mitigate heavy metal toxicity through growth promotion and enhanced antioxidant capacity [14,28]. Under heavy metal stress, reactive oxygen species (ROS) accumulate and cause lipid peroxidation, protein damage, and photosystem inhibition. BRs are known to upregulate antioxidant enzymes [29], including superoxide dismutase, catalase, and ascorbate peroxidase, as well as non-enzymatic antioxidants such as ascorbate and glutathione, thereby mitigating oxidative damage. The robust biomass maintenance under 24-Brs treatment despite elevated root Pb and As concentrations suggests that these stress tolerance pathways are actively activated, enabling plants to sustain growth at higher internal metal loads. Notably, there may be interactions between BR-mediated antioxidant defense and metal chelation pathways that are not necessarily synergistic but could be complementary: on the one hand, antioxidant enzymes scavenge metal-induced ROS to prevent oxidative damage; on the other hand, phytochelatins and metallothioneins directly bind free metal ions, reducing their toxicity and decreasing the source of ROS production. This multi-layered defense network may be the key mechanism by which 24-Brs maintains biomass despite high metal loads.

The magnitude of 24-Brs-mediated recovery differed significantly between the two species, reflecting intrinsic differences in baseline stress severity and, potentially, differences in BR signaling sensitivity. Vetiver grass showed a stronger biomass response than willow, which may be attributable to several factors. First, vetiver grass suffered more severe growth inhibition under Pb-As stress (21.31% vs. 12.44% for willow), leaving more room for recovery. Second, interspecific differences in BR signaling pathway sensitivity may play a role: as a C_4_ herbaceous plant, vetiver grass possesses a particularly robust BR-responsive growth regulatory network that supports rapid biomass accumulation under favorable hormonal conditions. Third, the root-centered metal retention strategy of vetiver may be more compatible with BR-mediated detoxification pathways, as root phytochelatin synthesis and vacuolar compartmentalization can be upregulated through BES1/BZR1-dependent transcription. Furthermore, the carbon-concentrating mechanism unique to C_4_ plants may provide more abundant energy and carbon skeleton support for BR-induced detoxification processes, enabling vetiver grass to invest more resources into metal detoxification pathways while maintaining high growth rates. This conjecture is consistent with the finding that vetiver grass showed the largest biomass increase (114.81% in monoculture) in this study.

When the two species were combined in intercropping, 24-Brs played an additional role in regulating interspecific interactions beyond its direct physiological effects. The intercropping system revealed asymmetric competition dynamics: willow biomass declined dramatically when co-planted with vetiver grass (from 41.25 to 7.26 g pot^−1^ in CK, *p* < 0.05), whereas vetiver grass was largely unaffected. Despite this competitive asymmetry, the total system biomass (vetiver + willow) under 24-Brs-WV (approximately 102.13 g) was comparable to that of V-Brs monoculture (103.56 g), indicating that 24-Brs partially alleviated the competitive suppression experienced by willow while enhancing stress tolerance in both species. This dual function, alleviating interspecific competition and enhancing metal resistance, is particularly valuable for phytoremediation systems, in which maintaining both species is crucial for complementary contaminant retention.

### 3.2. 24-Brs Regulates Pb and As Accumulation in a Species- and Tissue-Specific Manner

The effects of 24-Brs on Pb and As accumulation were complex, varying by element, plant species, tissue, and planting system. For willow, 24-Brs significantly increased root Pb (from 14.23 to 24.55 mg kg^−1^ in monoculture) while decreasing stem Pb (from 0.90 to 0.37 mg kg^−1^; a 58.89% reduction), a pattern consistent with enhanced root retention and reduced translocation to aboveground tissues. Leaf Pb did not change significantly under 24-Brs, indicating that the hormone primarily regulates root-to-stem transport rather than leaf-level accumulation.

This finding adds nuance to current perspectives on brassinosteroids and heavy metal accumulation. Previous studies have reported that 24-Brs reduces metal concentrations in plant tissues as part of its stress-alleviating mechanism [17,18], based primarily on crop systems in which reducing metal content in edible tissues is the primary goal. Our results indicate a more complex picture: in roots, 24-Brs can increase Pb concentrations (potentially beneficial for phytostabilization), whereas in aboveground tissues it reduces Pb concentrations (consistent with reduced toxicity). Both effects are favorable for phytoremediation: more metal immobilized in roots means stronger stabilization, and less translocation means lower risk of aboveground toxicity and safer harvest residues.

For As, 24-Brs moderately increased root As concentrations in both species (from 10.80 to 13.55 mg kg^−1^ in willow monoculture, a 25.46% increase; from 33.83 to 38.26 mg kg^−1^ in vetiver monoculture, a 13.09% increase), although the increases were not always statistically significant (all contaminated groups shared the same letter a). The smaller magnitude of change in As accumulation compared with Pb likely reflects the fact that As exists primarily in anionic forms in soil and is less affected by 24-Brs-induced root exudate cation mobilization effects and rhizosphere acidification. As uptake occurs mainly through phosphate transporters and aquaglyceroporins [30], which may be less responsive to BR signaling than the broad-spectrum cation transporters that mediate Pb uptake.

The interaction between 24-Brs and the planting pattern showed a moderate trend for root Pb in vetiver grass (*p* = 0.076): 24-Brs increased Pb concentration under intercropping (from 44.51 to 68.58 mg kg^−1^, a 54.08% increase), while slightly decreasing it in monoculture (from 55.56 to 53.49 mg kg^−1^), although neither simple effect reached conventional significance levels. We hypothesize that in the intercropped rhizosphere, the combined root systems create a more complex chemical environment, including diverse root exudates and microbial communities, that amplifies 24-Brs-mediated Pb mobilization. Willow roots may secrete organic compounds that chelate Pb and increase its bioavailability, and 24-Brs further enhances this effect by stimulating root exudation from both species. Intercropped vetiver grass, with its dense fibrous root system in the upper soil layers, then intercepts this mobilized Pb, leading to the observed concentration increase.

The low root-to-shoot translocation rates for Pb and As in both species confirm their primary function in phytostabilization rather than phytoextraction. 24-Brs enhanced root retention while further limiting upward translocation, a pattern consistent with enhanced chelation and vacuolar compartmentalization in root cells. For willow, known to phytoextract more mobile metals like Cd and Zn, this demonstrates that under Pb-As co-contamination, 24-Brs redirects its function toward phytostabilization.

To further quantify phytostabilization performance, bioconcentration factors (BCFs, the ratio of root metal concentration to total soil metal concentration) and translocation factors (TFs, the ratio of shoot to root metal concentration) were calculated. Under 24-Brs, willow root BCF for Pb reached 0.11 in monoculture and intercropped vetiver root BCF reached 0.32, confirming active root-level accumulation. TF values remained well below 1.0 across all treatments, indicating that 24-Brs-enhanced metal uptake was effectively contained within root tissues without increasing shoot translocation risk. These results confirm that both species function primarily in phytostabilization, and that 24-Brs strengthens this function by increasing root retention capacity.

### 3.3. Putative Physiological Mechanisms of BR-Enhanced Phytostabilization: Inferences from Physiological and Geochemical Data

The observed Pb accumulation pattern, increased root concentrations coupled with reduced stem and leaf translocation under 24-Brs treatment, is consistent with enhanced chelation and vacuolar compartmentalization in root cells [31]. This pattern suggests that 24-Brs may promote the synthesis of metal-chelating compounds such as phytochelatins and metallothioneins [32], which bind Pb in the cytosol and facilitate its transport to vacuoles for safe storage, thereby limiting upward transport to aboveground tissues. Meanwhile, enhanced cell wall binding of Pb in root endodermal tissue may contribute to reduced movement through the apoplastic pathway into the stele [33]. The result is greater Pb retention in root tissues, consistent with the phytostabilization goal of immobilizing contaminants belowground.

The differential responses of mineral nutrients to 24-Brs are consistent with the hypothesis that transporter-level regulation may contribute to BR-mediated metal homeostasis, although direct evidence is lacking. The contrasting patterns observed (Zn and Cu maintained at higher levels while Mn concentrations decreased sharply under 24-Brs) could potentially reflect differential regulation of distinct transporter families, if such regulation occurs. Zinc and copper share divalent cation uptake pathways with Pb (e.g., broad-spectrum cation transporters [34]), and their co-accumulation pattern suggests that 24-Brs may upregulate these broad-spectrum transporters, which inadvertently increases Pb uptake while fulfilling their primary function in mineral nutrition. In contrast, manganese is primarily influenced by soil redox changes rather than direct transporter regulation, as discussed in detail in Section 4.5.

The marked increase in soil oxidation–reduction potential (ORP) induced by 24-Brs indicates an indirect, rhizosphere-mediated mechanism of action whose effects extend beyond direct plant physiological effects. BRs are known regulators of root architecture and rhizosphere microbial interactions [35], promoting lateral root formation and root hair development, which could potentially increase root exudation and alter the rhizosphere microbial community [36], although root exudates and microbial communities were not directly measured in the present study. These changes in root exudate composition and microbial activity can modify soil redox conditions by altering oxygen consumption, organic matter turnover, and microbial respiration rates. The ORP increase observed in this study (as high as 95.12% for W-Brs relative to W-NBrs) indicates that 24-Brs pushes the rhizosphere toward more oxidizing conditions, with cascading effects on metal speciation that are explored in detail in Section 4.4 and Section 4.5.

### 3.4. 24-Brs Reshapes Soil Metal Speciation to Increase Bioavailability

Tessier sequential extraction results provide insights into the soil biogeochemical mechanism by which 24-Brs enhances metal uptake. Most notably, 24-Brs consistently and significantly increased the exchangeable fraction of Pb across different planting patterns (*p* < 0.05), while the effect on exchangeable As was not statistically significant, while significantly reducing the organic matter-bound fraction of Pb in V and WV treatments (*p* < 0.05). These fractionation shifts indicate that 24-Brs application mobilizes metals from less available pools into the exchangeable phase, making them more accessible for root uptake.

The increase in EX-Pb was dramatic (up to 63.22% in willow soil, 118.37% in vetiver soil). Since exchangeable metals are the most bioavailable fraction [37], this provides a geochemical mechanism for 24-Brs-enhanced plant metal accumulation. The concurrent decrease in OM-Pb suggests that 24-Brs promotes the release of organically bound metals into more bioavailable pools, potentially through enhanced root exudation or altered microbial activity in the rhizosphere [38].

Second, 24-Brs may alter the quantity and composition of root exudates [39]. By promoting lateral root formation and root hair development, 24-Brs expands the root surface area available for secretion, increasing the release of low-molecular-weight organic acids, phytosiderophores, and mucilaginous substances that can chelate metal ions and displace them from soil exchange sites [40]. The significant reduction in OM-Pb in V and WV treatments after 24-Brs application is consistent with this mechanism: root exudates may compete with soil organic matter for Pb binding sites, or they may stimulate microbial activity that mineralizes organic complexes and releases bound Pb. The concomitant decrease in EC under 24-Brs (32.91% decline in V-Brs vs. V-NBrs) is also noteworthy. Reduced electrical conductivity reflects lower soil solution ionic strength, which seems paradoxical given the increase in EX-Pb. However, this can be explained by enhanced plant uptake of mobilized cations, including Pb, Zn, and base cations, and increased microbial immobilization of solutes, such that solution ionic strength decreases despite metals being actively desorbed from the solid phase [41]. It should be noted, however, that root exudates and rhizosphere microbial communities were not directly measured in the present study, and the above interpretation is based on indirect evidence.

Third, the effect of 24-Brs on soil redox potential adds another layer of complexity. 24-Brs consistently and significantly increased ORP compared with NBrs, with increases of 60.20–95.12% in monoculture systems (*p* < 0.05). Higher ORP stabilizes Fe(III) and Mn(III/IV) oxides, which have high adsorption capacities for both Pb and As [42], an effect that would tend to decrease rather than increase exchangeable metal concentrations. This apparent contradiction can be resolved by recognizing that multiple processes operate simultaneously: 24-Brs-induced pH reduction and exudate chelation mobilize Pb from organic and exchange sites, while at the same time, higher ORP promotes iron-manganese oxide formation that can recapture some of the mobilized metal. FMO-Pb in vetiver soil actually increased significantly under 24-Brs (V-Brs: 103 mg kg^−1^), consistent with the simultaneous enhancement of oxide adsorption and Pb mobilization. The fact that EX-Pb still increases despite higher FMO-Pb indicates that for Pb, the mobilizing forces (pH reduction, organic chelation) outweigh the immobilizing force (oxide adsorption). For As, higher ORP stabilizes arsenate (As(V)) relative to arsenite (As(III)), and As(V) is more strongly adsorbed by Fe oxides, which helps explain why EX-As increases far less than EX-Pb: even under conditions that mobilize cationic metals, the oxide adsorption barrier effectively limits As migration [43].

For As, fractionation changes were more modest, consistent with the strong affinity of As for iron oxide surfaces [44]. The moderate increase in EX-As in willow soil under 24-Brs (from 3.80 to 4.20 mg kg^−1^, a 10.53% increase), although not statistically significant at the overall level, likely reflects the same chelation and pH mechanisms described above, although the magnitude is constrained by the tendency of As to remain adsorbed on Fe-Mn oxides even under moderately altered conditions. The elevated ORP under 24-Brs further limits As mobility by stabilizing As(V) and Fe(III) oxide surfaces, creating a geochemical buffer that prevents EX-As from increasing to the same extent as EX-Pb.

The fractionation results highlight a key conceptual point: 24-Brs is not merely a ‘growth promoter’ that makes plants larger while metal concentrations per unit mass remain unchanged. Rather, it actively modifies the rhizosphere environment, shifting metals between geochemical pools and thereby altering their bioavailability. This soil-mediated effect adds an important dimension to our understanding of how brassinosteroids influence plant–metal interactions, and suggests that 24-Brs-assisted phytoremediation operates through a combination of direct plant physiological effects and indirect soil biogeochemical effects.

The differential responses of Pb and As to 24-Brs application, both in soil fractions and plant accumulation, reveal element-specific mechanisms reflecting fundamental differences in their geochemical behavior and plant uptake pathways. For the divalent cation Pb, 24-Brs induced a large increase in exchangeable forms (up to 118.37% EX-Pb in vetiver soil), accompanied by significant increases in root Pb concentrations (72.54% in willow monoculture, 54.08% in intercropped vetiver). The strong coupling between soil EX-Pb mobilization and root Pb accumulation indicates that Pb, as a cation, readily enters root cells through broad-spectrum cation transporters that may be modulated by BR signaling [45], although this remains to be directly confirmed. The corresponding decrease in OM-Pb, driven by 24-Brs-mediated pH reduction and rhizosphere acidification, effectively displaces Pb from organic binding sites, releasing it into the soil solution for uptake.

In contrast, As exists in soil primarily as anionic oxyanions (arsenate and arsenite), and its geochemical behavior is dominated by strong adsorption to Fe-Mn oxide surfaces rather than organic matter complexes [46]. Consequently, 24-Brs only moderately increased EX-As (10.53% in willow soil), and changes in plant As accumulation were correspondingly smaller (root As increases of 13.09–25.46%, many not statistically significant). This mild response reflects two constraints: first, the high affinity of arsenate for Fe oxide surfaces limits the achievable magnitude of mobilization [47]; and second, As uptake pathways (phosphate transporters, aquaglyceroporins) are less directly responsive to BR signaling than broad-spectrum cation transporters.

The differential responses of Pb and As to 24-Brs reflect fundamental differences in their geochemistry. For Pb (divalent cation), 24-Brs induced large EX-Pb increases coupled with significant root accumulation, consistent with enhanced bioavailability through broad-spectrum cation transporters potentially modulated by BR signaling. For As (anionic oxyanion), changes were more modest due to its strong affinity for Fe-Mn oxide surfaces rather than organic matter complexes.

In contrast to Pb, As exists primarily as anionic oxyanions with strong adsorption to Fe-Mn oxide surfaces. Consequently, 24-Brs only moderately increased EX-As, and plant As accumulation changes were correspondingly smaller. The 24-Brs-induced ORP elevation further limits As mobility by stabilizing As(V) and promoting Fe(III) oxide formation, both of which enhance As retention in soil [48].

The redox context further differentiates element-specific behavior: higher ORP stabilizes Fe(III)/Mn(III,IV) oxides that adsorb As but immobilize Mn, while having more complex effects on Pb bioavailability. These element-specific responses demonstrate that 24-Brs does not simply increase overall metal bioavailability but rather reshapes the rhizosphere chemical environment in element-selective ways.

### 3.5. 24-Brs Differentially Affects Mineral Nutrient Dynamics Under Pb-As Stress

The Pb-As fractionation shifts discussed in Section 4.4 represent the major biogeochemical effects of 24-Brs, but they are not the only ones. 24-Brs also reshapes the speciation and uptake of essential mineral nutrients, changes that carry their own physiological and ecological significance. The five elements (Mn, Zn, Cu, Ni, Ca) exhibited distinctly different response patterns, forming a gradient from strong immobilization to significant increase that reflects each element’s unique chemical properties and interactions with plant regulatory networks.

Manganese showed the most dramatic response to 24-Brs: plant tissue concentrations declined sharply (up to 73.90% reduction in willow leaves), driven primarily by soil geochemistry rather than plant regulation. Soil fractionation showed 24-Brs significantly decreased EX-Mn while increasing FMO-Mn across all planting patterns, consistent with 24-Brs-induced ORP elevation promoting Mn oxide precipitation [49]. Nickel exhibited a more complex pattern, with the most pronounced intercropping effects suggesting species-specific rhizosphere interactions beyond simple BR-mediated regulation.

Zinc exhibited the opposite pattern to Mn: concentrations increased under contamination and 24-Brs maintained elevated levels, likely because Zn shares broad-spectrum cation transporters with Pb and is less affected by redox changes [50,51]. Copper, regulated by more specialized homeostatic mechanisms (potentially involving COPT transporters and CTR signaling [52,53]), showed intermediate behavior; 24-Brs partially reversed stress-induced accumulation, particularly in willow roots. Both Zn and Cu responses are consistent with the hypothesis that BR signaling modulates transporter activity, though direct verification is needed.

Calcium, a macronutrient and secondary messenger, responded differently from the redox-sensitive micronutrients. 24-Brs substantially increased root Ca in intercropped willow, potentially supporting cell wall development and Ca-mediated stress signaling, a physiological response largely decoupled from soil geochemical changes.

The differential responses of Mn, Zn, Cu, Ni, and Ca to 24-Brs revealed a clear gradient from highly redox-sensitive (Mn, strongly immobilized by 24-Brs-induced ORP elevation) to primarily physiologically regulated (Ca, controlled by signaling rather than soil geochemistry). Intermediate elements (Zn, Cu, Ni) exhibited mixed responses reflecting the interplay of soil geochemistry and species-specific transporter regulation. These patterns are consistent with each element’s chemical properties but require molecular-level validation.

### 3.6. Comparative Analysis of 24-Brs Effects Across Different Plant–Metal Systems

To place our findings in a broader context, it is informative to compare the magnitude and direction of 24-Brs effects observed in this study with those reported for other plant–metal combinations [54]. This cross-study comparison reveals several consistent patterns as well as some important differences, highlighting the context-dependence of BR-mediated metal stress responses.

In terms of biomass enhancement, our results are at the higher end of the range reported in the literature. A meta-analysis covering 73 studies reported an average 47.90% increase in shoot and root length after 24-Brs application [18], whereas we observed even stronger biomass increases of 63.89–114.81%. This stronger response likely reflects the use of woody and herbaceous perennials under significant Pb-As stress, conditions under which brassinosteroid-mediated growth promotion has greater scope for improvement compared with unstressed crop systems [55,56].

The direction of 24-Brs effects on metal accumulation shows greater variation. Our finding of increased root Pb and As concentrations contrasts with the 32.90% average reduction in tissue metal concentrations reported by the aforementioned meta-analysis [18]. However, this discrepancy is more apparent than real: the meta-analysis average is heavily biased toward crop species in which reducing metal accumulation (protecting edible tissues) is the primary goal, whereas our study focuses on phytoremediation species in which increased root retention is the desired outcome. In studies specifically targeting phytoremediation species, 24-Brs has been shown to increase metal accumulation: the combined application of 24-Brs with phosphate-solubilizing bacteria increased As accumulation in Sedum lineare by 97.30% [20]; a study on willow–alfalfa intercropping found that 24-Brs increased Cd extraction by 80.60% (from 0.31 to 0.56 mg pot^−1^) through combined effects on biomass and available Cd content [24]; and biochar-amended willow systems showed enhanced Cd phytoremediation with differentiated microbial community responses [57].

The pattern of 24-Brs effects also differs systematically among metals. For cationic metals (Pb, Cd, Zn, Cu), the effect of 24-Brs on accumulation is strongly concentration-dependent and tissue-specific: at low to moderate exposure levels, 24-Brs tends to increase root accumulation (through enhanced transporter expression and chelation) while reducing aboveground levels (through compartmentalization and reduced translocation). For anionic metalloids (As, Cr), effects typically include moderate increases in root accumulation, reflecting different uptake pathways (As via phosphate transporters, Cr via sulfate transporters) [58,59] and the fact that these metalloids participate in BR signaling networks through different entry points. The meta-analysis [18] indicates that effect sizes vary by metal type, with 24-Brs exerting stronger alleviating effects on Cd and Pb than on Cr and Cu.

There are also consistent differences in 24-Brs responsiveness across plant families. Brassicaceae plants typically exhibit reduced metal accumulation under BR treatment, consistent with their role as food crops [15]; Poaceae (including vetiver) tend to exhibit strong growth responses with moderate changes in metal accumulation; and woody species (Salicaceae) show strong root accumulation with enhanced phytostabilization, as observed in our willow. These differences have practical implications for species selection in 24-Brs-assisted phytoremediation [14,15,18,60].

## 4. Materials and Methods

### 4.1. Soil Collection and Preparation

The test soil was collected from the topsoil (0–20 cm) of a naturally contaminated agricultural field in Xushe Village, Jiangsu Province, China. The site has a contamination history associated with lead–zinc mining, and the soil profile contains naturally occurring Pb and As (the soil is naturally contaminated, not artificially spiked). Uncontaminated control soil was collected from an agricultural field approximately 2 km from the contaminated site, with a similar soil type and land use history but without metal contamination. After air-drying and passing through a 2 mm sieve, the contaminated soil was found to contain 215.48 mg Pb kg^−1^ and 120.16 mg As kg^−1^, whereas the uncontaminated control soil contained 22.62 mg Pb kg^−1^ and 10.21 mg As kg^−1^. The soil was thoroughly mixed and stored at room temperature in the dark prior to the pot experiment. Compared with artificially spiked soils, such naturally contaminated soils provide a more ecologically relevant substrate for phytoremediation testing, as they reflect the long-term weathering and complex fractionation patterns typical of field-contaminated sites. This study constitutes a rhizosphere soil investigation focusing on plant–root–soil interactions under natural contamination conditions.

To account for spatial heterogeneity within the contaminated site, soil was collected from five sub-sites distributed across the field in a grid pattern (approximately 10 m spacing between sub-sites). At each sub-site, fifteen soil cores (5.0 cm diameter, 0–20 cm depth) were collected using a stainless steel auger, then combined into a single composite sample. The five composite samples were further bulked together, thoroughly mixed, and homogenized by coning and quartering to produce a single representative composite sample of approximately 45 kg. This composite sampling strategy minimizes the influence of localized metal hotspots and ensures that the experimental soil reflects the average contamination level of the field.

Prior to homogenization, sub-samples from each of the five sub-sites were analyzed separately to assess spatial variability. The homogenized composite sample was then air-dried, passed through a 2 mm sieve, and characterized for initial physicochemical properties. The measured characteristics were: pH 6.30 ± 0.06, electrical conductivity (EC) 0.40 ± 0.03 mS/cm, oxidation–reduction potential (ORP) 248 ± 12 mV, and organic matter content 28.4 ± 1.6 g/kg (determined by loss-on-ignition at 550 °C for 4 h). The soil texture was classified as loam with 38.2% sand, 45.6% silt, and 16.2% clay. The cation exchange capacity (CEC) was 15.8 ± 1.2 cmol/kg. Total Fe and Mn concentrations were 32.4 ± 2.1 g/kg and 485 ± 28 mg/kg, respectively. Amorphous Fe and Mn (extracted with ammonium oxalate) were 2.85 ± 0.18 g/kg and 52.3 ± 4.1 mg/kg, respectively. Available phosphorus (Olsen method) was 12.6 ± 1.4 mg/kg. The initial soil also had total Pb of 215.48 ± 4.03 mg/kg and total As of 120.16 ± 7.41 mg/kg. All values are means ± standard deviation (*n* = 5 sub-site composites). The uncontaminated control soil, collected using the same composite sampling procedure from a clean field approximately 2 km away, had: pH 6.78 ± 0.08, EC 0.28 ± 0.02 mS/cm, organic matter 31.2 ± 2.1 g/kg, total Pb 22.62 ± 1.84 mg/kg, and total As 10.21 ± 0.93 mg/kg.

The moderate CV values for Pb and As across sub-sites are consistent with those reported for naturally contaminated paddy soils in the region, where historical mining runoff and atmospheric deposition create spatially heterogeneous but not extreme contamination gradients. Composite sampling effectively reduces this variability to levels that are negligible for pot-scale experimentation, ensuring that treatment effects can be reliably attributed to the experimental factors rather than to initial soil heterogeneity.

### 4.2. Plant Materials and Experimental Design

Willow preparation: Willow cuttings (NJU513, *Salix babylonica* × *S.* × *leucopithecia* ‘NJU513’) of approximately 10 cm in length with uniform diameter were selected, pre-rooted in half-strength Hoagland nutrient solution for 2 weeks, and then transplanted. Only cuttings that developed healthy roots and showed uniform initial growth were used in the experiment. Willow is widely used in phytoextraction and phytostabilization trials due to its rapid growth and deep, extensive root system [61].

Vetiver preparation: Seeds of vetiver grass (*Chrysopogon zizanioides*) were surface-sterilized and germinated on moist filter paper in Petri dishes. After germination, seedlings were transferred to half-strength Hoagland nutrient solution and cultured for an additional 2 weeks. Seedlings of consistent height and vigor were selected for transplantation. Vetiver grass is known for its strong tolerance to multiple heavy metals and its dense root system, which is suitable for phytostabilization.

A full-factorial pot experiment with a completely randomized design was conducted, comprising three planting patterns and three contamination–hormone treatments, with four replicates per treatment (Table 1). The planting patterns were: (W) willow monoculture (one willow cutting per pot), (V) vetiver grass monoculture (four vetiver seedlings per pot), and (WV) willow–vetiver intercropping (one willow cutting + four vetiver seedlings per pot). The treatment groups were: (planted CK) uncontaminated soil + plants (as the plant growth baseline), (NBrs) Pb-As contaminated soil + plants (no 24-Brs, contamination control), and (Brs) Pb-As contaminated soil + plants + 24-Brs application. An unplanted contaminated soil control (no-plant CK) was also included for soil property comparison. Each pot was filled with 1 kg of prepared soil.

Each pot contained 1.0 kg of soil (inner diameter 12 cm, height 15 cm). Willow monoculture pots contained one willow cutting; vetiver monoculture pots contained four vetiver seedlings; intercropping pots contained one willow cutting plus four vetiver seedlings. The unplanted contaminated control (CK without plants) was included for baseline comparison but was excluded from statistical analyses of plant parameters. The pot served as the experimental unit for soil measurements, and individual plants within each pot served as the experimental unit for plant measurements (*n* = 4 biological replicates per treatment).

24-Epibrassinolide (24-Brs; Sigma-Aldrich, St. Louis, MO, USA, purity ≥ 90%) was dissolved in deionized water and diluted to a final concentration of 2.08 μM, following the method of Li et al. (2023) [24]. 24-Brs was applied as a foliar spray every 7 days starting 15 days after transplantation, for approximately 16 applications during the 4-month growth period. The spray volume was determined by the point at which droplets naturally dripped from the leaf surface (plants were sprayed until droplets naturally fell from the leaves). Control plants were sprayed with deionized water. All plants were grown under natural greenhouse light (photoperiod approximately 12–14 h, PPFD approximately 400–600 μmol m^−2^ s^−1^ at midday) at day/night temperatures of 25–30/18–22 °C, and soil water content was maintained at 60–70% of field capacity by periodic weighing and replenishment with deionized water. Pots were re-randomized weekly within the greenhouse to minimize positional effects. The entire pot experiment lasted 4 months.

### 4.3. Leachate Collection

To evaluate the effectiveness of the willow–vetiver intercropping system in reducing Pb and As leaching, a simulated acid-rain event was conducted to examine the mobility of these two elements. The acid rain solution was prepared at pH 5.0 by mixing dilute H_2_SO_4_ (0.46 mg L^−1^) and HNO_3_ 0.69 mg L^−1^) stock solutions at a volume ratio of 6:4, yielding final sulfate and nitrate concentrations of approximately 0.27 mg L^−1^ and 0.27 mg L^−1^, respectively. This anion ratio reflects the typical composition of acid precipitation in southern China, where sulfate and nitrate are the dominant acidifying ions. The pH 5.0 value was selected to represent moderate acid rain conditions typical of agricultural regions in southern China, where rainfall pH values between 4.0 and 5.6 have been frequently documented in local environmental monitoring reports. Prior to the leaching test, the soil water content in the pots was adjusted to approximately 60% using a moisture probe. The acid solution was injected into the soil via a peristaltic pump at a rate of 0.613 mL min^−1^, with a cumulative total volume of 884 mL applied over 24 h. This volume corresponds to a rainfall depth of 78 mm which simulates a single heavy rainfall event based on local meteorological records for the Xushe region, where mean monthly precipitation during the rainy season (June–August) ranges from 150 to 200 mm and individual storm events frequently exceed 40 mm within 24 h. The permeating solution (initial pH 5.0) was collected at the bottom of each pot using a vacuum extractor. We acknowledge that a single leaching event cannot fully represent long-term contaminant mobility under natural rainfall conditions, and this limitation should be considered when interpreting the leachate results.

### 4.4. Plant and Soil Sample Collection

After 4 months of growth, all plants were harvested and separated into roots, stems, and leaves (willow) or roots and shoots (vetiver grass). Root samples were carefully rinsed with tap water to remove adhering soil particles, then washed with deionized water and blotted dry. Roots were rinsed three times with tap water followed by a final rinse with deionized water. Roots were then soaked in 0.01 M HNO_3_ for 30 min to remove adsorbed metal ions from the root surface, and subsequently blotted dry. This washing protocol, adapted from the standard root-washing procedure for metal-contaminated plants [62], was adopted to minimize external contamination while acknowledging that some surface-associated Pb may remain; therefore, root Pb values should be interpreted as operationally defined total root-associated Pb. After recording the fresh weight, the samples were dried at 70 °C to a constant weight for dry biomass determination. Rhizosphere soil was obtained by gently shaking the roots and collecting the soil that remained adhered to the roots [63]. Bulk soil samples were also collected from each pot. All soil samples were air-dried, ground, and passed through 0.149 mm and 2 mm sieves, respectively, for different analyses.

### 4.5. Soil Physicochemical Analysis

Soil pH was measured using a pH meter (PHS-3C, Leici, INESA Scientific Instrument Co., Ltd., Shanghai, China) in a 1:2.5 (*w*/*v*) soil–water suspension. The oxidation–reduction potential (ORP) was determined in the same suspension using an ORP electrode. Electrical conductivity (EC) was measured using a conductivity meter (DDS-11A, Leici, INESA Scientific Instrument Co., Ltd., Shanghai, China) in a 1:5 (*w*/*v*) soil–water extract.

### 4.6. Sequential Extraction of Metal Fractions

The Tessier sequential extraction procedure was used to determine the chemical fractions of Pb, As, Zn, Cu, Ni, and Mn in soil, dividing the metals into five operationally defined fractions: exchangeable (EX), carbonate-bound (CAB), Fe/Mn oxide-bound (FMO), organic matter-bound (OM), and residual [64]. The first four fractions (EX, CAB, FMO, OM) were quantified and reported in this study. The Tessier method remains one of the most widely used sequential extraction schemes for assessing metal partitioning in soils and sediments. The BCR three-step extraction method, another widely adopted standardized protocol, also provides an important methodological basis for metal fractionation analysis [65]. The residual fraction of heavy metals has no effect on plant heavy metal uptake and showed no significant difference among treatments; therefore, the residual fraction of heavy metals is not shown in the figures of this study.

Although the Tessier procedure was originally developed for cationic metals, it has been adapted and applied to arsenic fractionation in numerous soil studies [66]; for As, the Fe/Mn oxide-bound fraction is particularly important given the strong affinity of arsenate for iron oxide surfaces [64,65].

Briefly, 1.00 g of air-dried soil was weighed into 50 mL centrifuge tubes and subjected to the following extraction steps:

Exchangeable (EX): 8 mL of 1 M MgCl_2_ (pH 7.00), shaken at room temperature for 1 h.

Carbonate-bound (CAB): 8 mL of 1 M NaOAc (pH 5.00, adjusted with HOAc), shaken for 5 h.

Fe/Mn oxide-bound (FMO): 20 mL of 0.04 M NH_2_OH·HCl (in 25% (*v*/*v*) HOAc), heated at 96 ± 3 °C for 6 h with occasional agitation.

Organic matter-bound (OM): 3 mL of 0.02 M HNO_3_ + 5 mL of 30% H_2_O_2_ (pH 2.00), heated at 85 °C for 2 h, followed by an additional 3 mL of H_2_O_2_ and heating for 3 h; subsequently, 5 mL of 3.2 M NH_4_OAc (in 20% (*v*/*v*) HNO_3_) was added and the mixture was shaken for 30 min.

After each extraction step, the samples were centrifuged at 4000 rpm for 15 min, and the supernatant was filtered and collected for analysis. Between extraction steps, the residues were washed with 8 mL of deionized water. All extractions were performed in triplicate, and each measurement was averaged before statistical analysis.

### 4.7. Analysis of Total Metal Concentrations in Plants and Soil

Dried plant samples (0.20 g) were digested with HNO_3_–HClO_4_ (4:1, *v*/*v*) in an electric heating digestion system. Total metal concentrations in soil were determined by digesting 0.50 g of soil with HF–HNO_3_–HClO_4_ [67]. The concentrations of Pb, As, Zn, Cu, Ni, Mn, and Ca in all digests and extracts were measured using inductively coupled plasma optical emission spectrometry (ICP-OES, Agilent 720ES, Santa Clara, CA, USA). Standard reference materials (GBW07405 for soil, GBW07603 for plants) were included in each batch of analysis for quality control, with recovery rates ranging from 92% to 108%, meeting accepted quality assurance standards for trace element analysis. The method detection limits (MDLs) were 3.20 μg L^−1^ for Pb and 1.50 μg L^−1^ for As. Recoveries of certified reference materials ranged from 94.2% to 103.8% for Pb and from 91.5% to 105.3% for As, with calibration curves showing R^2^ > 0.999 and instrument drift checked every 10 samples.

### 4.8. Statistical Analysis

All data were subjected to normality tests (Shapiro–Wilk test) and homogeneity of variance tests (Levene’s test) prior to statistical analysis. Two-way analysis of variance (ANOVA) was performed to evaluate the main effects of planting pattern and 24-Brs treatment, as well as their interaction, followed by Tukey’s honestly significant difference (HSD) test to compare means among treatments at the *p* < 0.05 significance level (Supplementary Materials). Different lowercase letters above bars in the figures indicate significant differences among treatments. All statistical analyses were performed using SPSS 22.0 (IBM, Armonk, NY, USA), and graphs were plotted using OriginPro 2023b (OriginLab Corporation, Northampton, MA, USA). Data in tables and figures are presented as the mean ± standard deviation (*n* = 4).

## 5. Conclusions

This study provides a comprehensive analysis of how 24-epibrassinolide (24-Brs) regulates plant–metal–soil interactions in willow–vetiver intercropping systems under Pb-As co-contamination. 24-Brs significantly alleviated Pb-As phytotoxicity and stimulated plant growth, with biomass increases of 63.89–114.81% across species and planting patterns relative to the stress control (*p* < 0.05), with vetiver grass showing a stronger growth response than willow. The hormone significantly enhanced plant root Pb accumulation (most notably a 72.54% increase in monoculture willow roots and a 54.08% increase in intercropped vetiver roots, *p* < 0.05) while reducing Pb translocation to aboveground organs, thereby enhancing phytostabilization efficiency.

Soil fractionation analysis demonstrated that 24-Brs mobilized Pb and As from less bioavailable pools (particularly organic matter-bound Pb) into the exchangeable fraction (EX-Pb increased by up to 118.37%, *p* < 0.05), providing a soil biogeochemical mechanism for enhanced metal bioavailability. The hormone also reshaped the fractions of essential mineral nutrients (Zn, Cu, Ni, Mn) in element-specific patterns, maintaining higher Zn and Cu levels to support stress tolerance while substantially reducing Mn uptake, an effect associated with 24-Brs-induced elevation of soil redox potential that promotes Mn oxide formation. The willow–vetiver intercropping system achieved the lowest As leaching (1.50 μg L^−1^ with Brs-WV, a 90.20% reduction compared with the unplanted control), although the reduction by 24-Brs within each planting pattern was not statistically significant, demonstrating strong contaminant immobilization capacity achieved through complementary rooting strategies and rhizosphere interactions.

These results indicate that 24-Brs enhances Pb-As phytostabilization through combined effects on plant metal accumulation patterns and soil biogeochemistry. While the observed changes in plant metal accumulation and soil metal fractionation are consistent with enhanced root retention and metal mobilization, respectively, the underlying plant physiological and molecular mechanisms remain to be directly investigated. The willow–vetiver–24-Brs system represents a cost-effective and environmentally sustainable strategy for the in situ remediation of Pb-As co-contaminated soils, particularly applicable to tailings, industrial sites, and contaminated riparian zones in which phytostabilization is the primary remediation objective.

## Figures and Tables

**Table 1:** Experimental design protocol for the pot experiment.

Treatments	W	V	WV		Pb (mg kg^−1^)	As (mg kg^−1^)
CK with plants	Willow	Vetiver	Willow	Vetiver	22.62	10.21
CK without plants	--	--	--	--	215.48	120.16
Brs	Willow	Vetiver	Willow	Vetiver	215.48	120.16
NBrs	Willow	Vetiver	Willow	Vetiver	215.48	120.16

Note: “--” indicates that no plants are cultured.

## Data Availability

The datasets generated and/or analyzed during the current study are available from the corresponding author upon reasonable request.

## Supplementary Materials

The following supporting information can be downloaded at: https://www.mdpi.com/article/10.3390/plants15213281/s1, Figure S1: The residual fractions of soil As, Cu, Pb, Ni, Zn, and Mn. Table S1: Enrichment of copper (Cu), manganese (Mn), zinc (Zn), nickel (Ni), and calcium (Ca) in various parts of willow after application of 24 epibrassinolide (mg kg^−1^). Table S2: Enrichment of copper (Cu), manganese (Mn), zinc (Zn), nickel (Ni), and calcium (Ca) in various parts of vetiver after application of 24 epibrassinolide (mg kg^−1^). Table S3: Two-way ANOVA results for pot-level soil chemical properties and heavy metal fractions. Table S4: Two-way ANOVA results for willow plant data. Table S5: Two-way ANOVA results for vetiver plant data.

## References

[1] Li Z., Ma Z., van der Kuijp T.J., Yuan Z., Huang L. (2014). A review of soil heavy metal pollution from mines in China: Pollution and health risk assessment. Sci. Total Environ..

[2] Islam M.R., Saxena V., Sharma A. (2024). Phytoremediation as a green and sustainable prospective method for heavy metal contamination: A review. RSC Sustain..

[3] Sarwar N., Imran M., Shaheen M.R., Ishaque W., Kamran M.A., Matloob A., Rehim A., Hussain S. (2017). Phytoremediation strategies for soils contaminated with heavy metals: Modifications and future perspectives. Chemosphere.

[4] Raskin I., Smith R.D., Salt D.E. (1997). Phytoremediation of metals: Using plants to remove pollutants from the environment. Curr. Opin. Biotechnol..

[5] Kuzovkina Y.A., Volk T.A. (2009). The characterization of willow (*Salix* L.) varieties for use in ecological engineering applications: Co-ordination of structure, function and autecology. Ecol. Eng..

[6] Liu Y., Huang L., Wen Z., Fu Y., Liu Q., Xu S., Li Z., Liu C., Yu C., Feng Y. (2023). Effects of intercropping on safe agricultural production and phytoremediation of heavy metal-contaminated soils. Sci. Total Environ..

[7] Danh L.T., Truong P., Mammucari R., Tran T., Foster N. (2009). Vetiver grass, *Vetiveria zizanioides*: A choice plant for phytoremediation of heavy metals and organic wastes. Int. J. Phytoremediation.

[8] Abbas G., Murtaza B., Bibi I., Shahid M., Niazi N.K., Khan M.I., Amjad M., Natasha M. (2018). Arsenic uptake, toxicity, detoxification, and speciation in plants: Physiological, biochemical, and molecular aspects. Int. J. Environ. Res. Public Health.

[9] Janssen J., Weyens N., Croes S., Beckers B., Meiresonne L., Van Peteghem P., Carleer R., Vangronsveld J. (2015). Phytoremediation of metal contaminated soil using willow: Exploiting plant-associated bacteria to improve biomass production and metal uptake. Int. J. Phytoremediation.

[10] Pulford I.D., Watson C. (2003). Phytoremediation of heavy metal-contaminated land by trees A review. Environ. Int..

[11] Zhang C., Zha Y., Guang P., Ai F., Yin Y., Guo H. (2024). Synergistic effects of tree-herb intercropping on the phytoremediation efficiency of cadmium and lead contaminated soil. Sci. Total Environ..

[12] Jan S., Alyemeni M.N., Wijaya L., Alam P., Siddique K.H., Ahmad P. (2018). Interactive effect of 24-epibrassinolide and silicon on antioxidant enzymes and cadmium uptake in *Pisum sativum* L.. BMC Plant Biol..

[13] An Y., Wang Y., Tan S.N., Yusof M.L.M., Ghosh S., Chen Z. (2020). Phytoremediation: A promising approach for revegetation of heavy metal-polluted land. Front. Plant Sci..

[14] Emamverdian A., Ding Y., Alyemeni M.N., Barker J., Liu G., Li Y., Mokhberdoran F., Ahmad P. (2022). Benzylaminopurine and Abscisic Acid Mitigates Cadmium and Copper Toxicity by Boosting Plant Growth, Antioxidant Capacity, Reducing Metal Accumulation and Translocation in Bamboo [*Pleioblastus pygmaeus* (Miq.)] Plants. Antioxidants.

[15] Emamverdian A., Khalofah A., Pehlivan N., Zia-ur-Rehman M., Li Y., Zargar M. (2024). Exogenous application of jasmonates and brassinosteroids alleviates lead toxicity in bamboo by altering biochemical and physiological attributes. Environ. Sci. Pollut. Res..

[16] Corso M., García de la Torre V.S. (2020). Biomolecular approaches to understanding metal tolerance and hyperaccumulation in plants. Metallomics.

[17] Bajguz A., Hayat S. (2009). Effects of brassinosteroids on the plant responses to environmental stresses. Plant Physiol. Biochem..

[18] Niu K., Zhang Y., Li S., Xiao H., Wang Y., Cui T., Zhao C. (2024). A meta-analysis on plant growth and heavy metals uptake with the application of 2,4-epibrassinolide in contaminated soils. Ecotoxicol. Environ. Saf..

[19] Rao I.M., Dixon R.A. (2017). Brassinosteroid mediated cell wall remodeling in grasses under abiotic stress. Front. Plant Sci..

[20] Yang S., Chu Y., Liu Z., Zhang C., Yu Y., Wang J. (2026). Synergistic improvement of phytoremediation efficiency of arsenic-contaminated soil with *Sedum lineare* by phosphate-solubilizing strain P-1 and 2,4-epibrassinolide. J. Environ. Sci..

[21] Sharma A., Kumar V., Shahzad B., Ramakrishnan M., Yuan H., Thukral A.K., Bhardwaj R., Zheng B. (2022). Abiotic stress tolerance in plants: Brassinosteroids navigate competently. Int. J. Mol. Sci..

[22] Alloway B.J. (2013). Heavy Metals in Soils: Trace Metals and Metalloids in Soils and Their Bioavailability.

[23] Ali H., Khan E., Ilahi I. (2019). Environmental chemistry and ecotoxicology of hazardous heavy metals: Environmental persistence, toxicity, and bioaccumulation. J. Chem..

[24] Li Y., Dong Q., Wu D., Liu F., Wang J., Li Z. (2023). A 24-epibrassinolide treatment and intercropping willow with alfalfa increase the efficiency of the phytoremediation of cadmium-contaminated soil. Sci. Total Environ..

[25] Song W., Li H., Zhao Z., Si R., Deng W., Wang M., Li Y. (2025). 24-Epibrassinolide Enhanced Plant Antioxidant System and Cadmium Bioavailability Under Soil Cadmium Stress. Plants.

[26] Rajewska I., Talarek M., Bajguz A. (2016). Brassinosteroids and response of plants to heavy metals action. Front. Plant Sci..

[27] Ortiz-Morea F.A., He P., Shan L., Russinova E. (2020). It takes two to tango—molecular links between plant immunity and brassinosteroid signalling. J. Cell Sci..

[28] Della Rovere F., De Domenico E., Francia P., Altamura C., Altamura M.M., Fattorini L. (2025). Brassinosteroids and jasmonates mitigate arsenite- and arsenate-induced stress. Plant Sci..

[29] Hasanuzzaman M., Bhuyan M.H.M.B., Zayed M., Ozturk M., Rahman M.A., Hossain M.A., Mahmud J.A., Fujita M. (2020). Reactive oxygen species and antioxidant defense in plants under abiotic stress: Revisiting the crucial role of a universal defense regulator. Antioxidants.

[30] Lilay G.H., Thiébaut N., de Bang T.C., Laursen K.H., Jørgensen K., Persson D.P., Husted S. (2024). Linking the key physiological functions of essential micronutrients to their deficiency symptoms in plants. New Phytol..

[31] Sharma S.S., Dietz K.-J., Mimura T. (2016). Vacuolar compartmentalization as indispensable component of heavy metal detoxification in plants. Plant Cell Environ..

[32] Cobbett C., Goldsbrough P. (2002). Phytochelatins and metallothioneins: Roles in heavy metal detoxification and homeostasis. Annu. Rev. Plant Biol..

[33] Krzesłowska M. (2011). The cell wall in plant cell response to trace metals: Polysaccharide remodeling and its role in defense strategy. Acta Physiol. Plant..

[34] Milner M.J., Seamon J., Craft E., Kochian L.V. (2013). Transport properties of members of the ZIP family in plants and their role in Zn and Mn homeostasis. J. Exp. Bot..

[35] Eichmann R., Richards L., Schäfer P. (2021). Hormones as go-betweens in plant microbiome assembly. Plant J..

[36] Vives-Peris V., de Ollas C., Gómez-Cadenas A., Pérez-Clemente R.M. (2020). Root exudates: From plant to rhizosphere and beyond. Plant Cell Rep..

[37] Violante A., Cozzolino V., Perelomov L., Caporale A.G., Pigna M. (2010). Mobility and bioavailability of heavy metals and metalloids in soil environments. J. Soil Sci. Plant Nutr..

[38] Wenzel W.W. (2009). Rhizosphere processes and management in plant-assisted bioremediation (phytoremediation) of soils. Plant Soil.

[39] Evangelou M.W.H., Ebel M., Schaeffer A. (2007). Chelate assisted phytoextraction of heavy metals from soil: Effect, mechanism, toxicity, and fate of chelating agents. Chemosphere.

[40] Ma W., Tang S., Dengzeng Z., Zhang D., Zhang T., Ma X. (2022). Root exudates contribute to belowground ecosystem hotspots: A review. Front. Microbiol..

[41] Hinsinger P., Bengough A.G., Vetterlein D., Young I.M. (2009). Rhizosphere: Biophysics, biogeochemistry and ecological relevance. Plant Soil.

[42] Borch T., Kretzschmar R., Kappler A., Van Cappellen P., Ginder-Vogel M., Voegelin A., Campbell K. (2009). Biogeochemical redox processes and their impact on contaminant dynamics. Environ. Sci. Technol..

[43] Masscheleyn P.H., Delaune R.D., Patrick W.H. (1991). Effect of redox potential and pH on arsenic speciation and solubility in a contaminated soil. Environ. Sci. Technol..

[44] Hao L., Liu M., Wang N., Li G. (2018). A critical review on arsenic removal from water using iron-based adsorbents. RSC Adv..

[45] Pourrut B., Shahid M., Dumat C., Winterton P., Pinelli E. (2011). Lead uptake, toxicity, and detoxification in plants. Rev. Environ. Contam. Toxicol..

[46] Miretzky P., Cirelli A.F. (2010). Remediation of arsenic-contaminated soils by iron amendments: A review. Crit. Rev. Environ. Sci. Technol..

[47] Bhattacharya P., Welch A.H., Stollenwerk K.G., McLaughlin M.J., Bundschuh J., Panaullah G. (2007). Arsenic in the environment: Biology and chemistry. Sci. Total Environ..

[48] Yamaguchi N. (2025). Mechanisms underlying abiotic immobilization of inorganic arsenic in flooded paddy soils: A review. Soil. Sci. Plant Nutr..

[49] Hu H., Li X., Gao X., Wang L., Li B., Zhan F., He Y., Qin L., Liang X. (2024). A review on the multifaceted effects of δ-MnO_2_ on heavy metals, organic matter, and other soil components. RSC Adv..

[50] Broadley M.R., White P.J., Hammond J.P., Zelko I., Lux A. (2007). Zinc in plants. New Phytol..

[51] Cakmak I. (2000). Role of zinc in protecting plant cells from reactive oxygen species. New Phytol..

[52] Yruela I. (2009). Copper in plants: Acquisition, transport and interactions. Funct. Plant Biol..

[53] Peñarrubia L., Romero P., Carrió-Seguí À., Andrés-Bordería A., Moreno J., Sanz A. (2015). Temporal aspects of copper homeostasis and its crosstalk with hormones. Front. Plant Sci..

[54] Kour J., Kohli S.K., Khanna K., Bakshi P., Sharma P., Singh A.D., Ibrahim M., Devi K., Sharma N., Ohri P. (2021). Brassinosteroid signaling, crosstalk and, physiological functions in plants under heavy metal stress. Front. Plant Sci..

[55] Anwar A., Liu Y., Dong R., Bai L., Yu X., Li Y. (2018). The physiological and molecular mechanism of brassinosteroid in response to stress: A review. Biol. Res..

[56] Yang C., Zhang C., Wang Z. (2024). Functions and mechanisms of brassinosteroids in regulating crop agronomic traits. Plant Cell Physiol..

[57] Di D., Wang S., Gai X., Xiao J., Li H., Chen G. (2026). Biochar enhanced phytoremediation efficiency of *Salix* for soil cadmium: The differentiated responses of microbial community. Biochar.

[58] Catarecha P., Segura M.D., Franco-Zorrilla J.M., García-Ponce B., Lanza M., Solano R., Paz-Ares J., Leyva A. (2007). A mutant of the Arabidopsis phosphate transporter PHT1;1 displays enhanced arsenic accumulation. Plant Cell.

[59] Xu Z.-R., Cai M.-L., Chen S.-H., Huang X.-Y., Zhao F.-J., Wang P. (2021). High-affinity sulfate transporter Sultr1;2 is a major transporter for Cr(VI) uptake in plants. Environ. Sci. Technol..

[60] Kidd P., Mench M., Álvarez-López V., Bert V., Dimitriou I., Friesl-Hanl W., Gallegos M., Guns M., Hijri M., Kumpiene J. (2015). Agronomic practices for improving gentle remediation of trace element-contaminated soils. Int. J. Phytoremediation.

[61] Jiang C., Wang Y., Chen Y., Wang S., Mu C., Shi X. (2024). The phytoremediation potential of 14 *Salix* clones grown in Pb/Zn and Cu mine tailings. Forests.

[62] Raskin I., Kumar P.B.A.N., Dushenkov V., Salt D.E. (1994). Bioconcentration of heavy metals by plants. Curr. Opin. Biotechnol..

[63] Bais H.P., Weir T.L., Perry L.G., Gilroy S., Vivanco J.M. (2006). The role of root exudates in rhizosphere interactions with plants and other organisms. Annu. Rev. Plant Biol..

[64] Tessier A., Campbell P.G.C., Bisson M. (1979). Sequential extraction procedure for the speciation of particulate trace metals. Anal. Chem..

[65] Rauret G., López-Sánchez J.F., Sahuquillo A., Rubio R., Davidson C., Ure A., Quevauviller P. (1999). Improvement of the BCR three step sequential extraction procedure prior to the certification of new sediment and soil reference materials. J. Environ. Monit..

[66] Wenzel W.W., Kirchbaumer N., Prohaska T., Stingeder G., Lombi E., Adriano D.C. (2001). Arsenic fractionation in soils using an improved sequential extraction procedure. Anal. Chim. Acta.

[67] Kabata-Pendias A. (2011). Trace Elements in Soils and Plants.

