# Supplementary material for: 24-Epibrassinolide Enhances Pb-As Phytostabilization in Willow-Vetiver Intercropping Systems: Plant Accumulation Patterns and Soil Biogeochemical Perspectives
Source: Plants (Basel). 2026 Oct 24;15(21):3281. doi: 10.3390/plants15213281 (PMC13766032; doi:10.3390/plants15213281)
Supplement: Supplementary file 1 [file plants-15-03281-s001.zip › plants-4590509-supplementary.pdf]

Table S1 Enrichment of copper (Cu), manganese (Mn), zinc (Zn), nickel (Ni), and calcium (Ca) in various parts of willow after application of 24-epibrassinolide (mg kg^{-1}).

Treatments		Roots (mg kg^{-1})		Stems(mg kg^{-1})		Leaves(mg kg^{-1})	
		W	WV	W	WV	W	WV
CK		2511±214^{c}	3565±571^{b}	13315±2293^{a}	13034±718^{a}	23116±818^{bc}	26934±3253^{a}
Brs	Ca	2695±227^{c}	4608±870^{a}	12027±952^{a}	12713±2493^{a}	23163±1211^{bc}	26494±3154^{ab}
NBrs		3037±311^{bc}	2933±167^{bc}	12926±617^{a}	11688±623^{a}	21977±1960^{c}	24060±2060^{abc}
CK		9.77±1.60^{b}	10.52±1.08^{b}	7.18±1.55^{c}	7.63±0.70^{c}	8.15±0.92^{bc}	6.46±0.75^{d}
Brs	Cu	14.08±2.74^{b}	14.03±0.97^{b}	7.32±0.46^{c}	9.44±2.31^{bc}	7.72±0.34^{cd}	7.09±0.42^{cd}
NBrs		31.77±10.60^{a}	16.74±4.98^{b}	12.93±3.37^{a}	11.65±0.69^{ab}	11.87±1.10^{a}	9.15±1.05^{b}
CK		54.06±14.17^{ab}	27.46±4.30^{c}	33.22±8.04^{a}	16.78±2.78^{b}	162.44±17.10^{a}	75.58±6.79^{c}
Brs	Mn	34.11±20.36^{bc}	16.41±2.92^{c}	9.21±1.78^{c}	8.22±1.26^{c}	42.40±2.42^{d}	38.09±2.94^{d}
NBrs		67.13±16.82^{a}	20.64±9.68^{c}	20.92±3.50^{b}	14.59±3.78^{bc}	104.92±20.29^{b}	47.04±6.75^{d}
CK		4.81±0.92^{b}	4.79±1.51^{b}	1.16±0.28^{a}	1.15±0.31^{a}	3.03±0.31^{b}	2.84±0.63^{b}
Brs	Ni	4.27±0.47^{b}	2.59±0.59^{b}	1.28±0.16^{a}	1.38±0.68^{a}	4.65±0.73^{a}	2.68±1.13^{b}
NBrs		11.02±4.34^{a}	3.62±0.40^{b}	1.54±0.12^{a}	1.23±0.16^{a}	4.42±0.43^{a}	3.83±0.82^{ab}
CK		72.27±4.39^{b}	83.82±2.64^{b}	93.50±11.40^{c}	114.42±9.24^{bc}	170.03±14.71^{c}	197.14±1.42^{bc}
Brs	Zn	84.91±5.09^{b}	104.40±9.87^{a}	118.85±6.94^{b}	152.80±26.97^{a}	208.33±12.31^{ab}	241.37±29.60^{a}
NBrs		105.50±7.69^{a}	98.95±11.66^{a}	144.55±9.62^{a}	160.26±4.04^{a}	229.62±22.39^{ab}	216.95±21.61^{ab}

Note: Different letters indicate significant differences (P< 0.05) in the enriched concentrations of the same element in the same plant tissue among different treatments.

Table S2 Enrichment of copper (Cu), manganese (Mn), zinc (Zn), nickel (Ni), and calcium (Ca) in various parts of vetiver after application of 24-epibrassinolide (mg kg^{-1}).

Treatments	Elements	Roots (mg kg^{-1})		Shoots (mg kg^{-1})	
		V	WV	V	WV
CK	Cu	$17.96±2.25^{b}$	$17.23±2.92^{b}$	$7.15±0.36^{b}$	$7.08±0.83^{b}$
Brs		$44.37±5.87^{a}$	$36.72±5.79^{a}$	$7.31±0.59^{b}$	$7.00±0.50^{b}$
NBrs		$43.63±6.90^{a}$	$39.13±7.03^{a}$	$10.03±1.05^{a}$	$9.33±1.27^{a}$
CK	Mn	$114.07±26.74^{a}$	$148.95±75.68^{a}$	$51.43±5.41^{ab}$	$52.25±16.44^{a}$
Brs		$171.21±9.42^{a}$	$146.93±79.17^{a}$	$45.65±6.05^{ab}$	$37.68±6.41^{b}$
NBrs		$172.68±48.25^{a}$	$191.96±46.46^{a}$	$42.98±6.17^{ab}$	$38.98±5.09^{ab}$
CK	Ni	$12.67±3.00^{c}$	$15.56±2.62^{c}$	$7.26±1.24^{b}$	$7.36±2.30^{b}$
Brs		$13.29±3.80^{c}$	$31.26±6.51^{b}$	$5.72±2.39^{bc}$	$3.72±0.81^{c}$
NBrs		$12.93±1.04^{c}$	$37.48±4.31^{a}$	$12.99±2.06^{a}$	$15.63±2.57^{a}$
CK	Zn	$74.55±6.16^{b}$	$89.26±10.59^{b}$	$73.12±5.58^{c}$	$78.15±9.36^{c}$
Brs		$125.40±18.77^{a}$	$142.22±10.84^{a}$	$109.29±10.33^{b}$	$110.47±10.05^{b}$
NBrs		$132.30±14.95^{a}$	$139.81±8.21^{a}$	$128.65±14.35^{a}$	$129.67±13.44^{a}$
CK	Ca	$1946±216^{a}$	$1841±117^{a}$	$7860±816^{b}$	$8275±237^{b}$
Brs		$1713±346^{a}$	$1072±409^{a}$	$9623±1263^{a}$	$10005±558^{a}$
NBrs		$1094±298^{b}$	$967±46^{b}$	$10054±959^{a}$	$9569±381^{a}$

Note: Different letters indicate significant differences ($p < 0.05$) in the enriched concentrations of the same element in the same plant tissue among different treatments.

Table S3. Two-way ANOVA results for pot-level soil chemical properties and heavy metal fractions

Variable	df_A	F_A	*P_A*	Sig.	df_B	F_B	*P_B*	Sig.	df_AB	F_AB	*P_AB*	Sig.
Leachate pH (before)	2	18.027	0.0001	***	1	0.476	0.5208	ns	2	3.266	0.0665	ns
Leachate pH (after)	2	22.665	< 0.0001	***	1	0.010	0.9250	ns	2	10.984	0.0012	**
Soil pH	2	113.463	< 0.0001	***	1	125.157	< 0.0001	***	2	16.715	0.0002	***
Soil EC	2	9.790	0.0019	**	1	4.119	0.0605	ns	2	0.656	0.5331	ns
Soil ORP	2	0.521	0.6041	ns	1	16.953	0.0009	***	2	1.693	0.2172	ns
Exchangeable Pb	2	67.699	< 0.0001	***	1	321.666	< 0.0001	***	2	8.470	0.0035	**
Carbonate-bound Pb	2	3.726	0.0486	*	1	1.399	0.2553	ns	2	1.783	0.2019	ns
Fe/Mn oxide-bound Pb	2	7.864	0.0046	**	1	2.561	0.1304	ns	2	3.095	0.0749	ns
Organic matter-bound Pb	2	0.029	0.9719	ns	1	2.006	0.1771	ns	2	0.882	0.4343	ns
Residual Pb	2	0.484	0.6253	ns	1	1.549	0.2324	ns	2	1.528	0.2489	ns
Exchangeable As	2	0.138	0.8719	ns	1	2.062	0.1716	ns	2	1.043	0.3766	ns
Carbonate-bound As	2	0.878	0.4359	ns	1	0.027	0.8714	ns	2	0.511	0.6101	ns
Fe/Mn oxide-bound As	2	0.978	0.3986	ns	1	0.067	0.7991	ns	2	0.865	0.4409	ns
Organic matter-bound As	2	1.978	0.1728	ns	1	3.140	0.0967	ns	2	3.316	0.0642	ns
Residual As	2	9.137	0.0025	**	1	34.415	< 0.0001	***	2	1.289	0.3043	ns
Exchangeable Zn	2	15.108	0.0003	***	1	147.781	< 0.0001	***	2	70.912	< 0.0001	***
Fe/Mn oxide-bound Zn	2	26.962	< 0.0001	***	1	0.586	0.4557	ns	2	2.963	0.0823	ns
Organic matter-bound Zn	2	2.088	0.1585	ns	1	2.890	0.1098	ns	2	2.348	0.1296	ns
Exchangeable Cu	2	1.780	0.2024	ns	1	0.003	0.9602	ns	2	4.813	0.0243	*
Fe/Mn oxide-bound Cu	2	2.644	0.1038	ns	1	0.342	0.5695	ns	2	1.260	0.3121	ns
Organic matter-bound Cu	2	3.403	0.0605	ns	1	3.874	0.0678	ns	2	4.252	0.0345	*
Exchangeable Ni	2	7.524	0.0076	**	1	0.001	0.9720	ns	2	0.378	0.6915	ns
Fe/Mn oxide-bound Ni	2	1.761	0.2057	ns	1	0.459	0.5082	ns	2	0.772	0.4796	ns
Organic matter-bound Ni	2	0.642	0.5402	ns	1	1.672	0.2155	ns	2	0.074	0.9289	ns
Exchangeable Mn	2	14.804	0.0003	***	1	55.983	< 0.0001	***	2	6.703	0.0083	**
Fe/Mn oxide-bound Mn	2	7.319	0.0061	**	1	8.934	0.0113	*	2	1.090	0.3613	ns
Organic matter-bound Mn	2	7.147	0.0066	**	1	5.454	0.0338	*	2	0.887	0.4325	ns
Carbonate-bound Mn	2	0.553	0.5867	ns	1	7.472	0.0154	*	2	14.386	0.0003	***
Residual Mn	2	0.704	0.5104	ns	1	16.581	0.0010	**	2	0.482	0.6269	ns
Carbonate-bound Zn	2	14.784	0.0003	***	1	0.109	0.7453	ns	2	10.729	0.0013	**
Residual Zn	2	1.395	0.2781	ns	1	60.042	< 0.0001	***	2	4.682	0.0263	*
Carbonate-bound Cu	2	1.745	0.2082	ns	1	0.678	0.4233	ns	2	2.005	0.1692	ns
Residual Cu	2	2.346	0.1299	ns	1	4.636	0.0480	*	2	2.335	0.1310	ns
Carbonate-bound Ni	2	8.418	0.0052	**	1	0.303	0.5901	ns	2	0.551	0.5875	ns
Residual Ni	2	1.936	0.1787	ns	1	1.423	0.250	ns	2	3.286	0.0655	ns

Note: Factor A = Planting mode (W, V, WV); Factor B = 24-Epi treatment (Brs, NBrs). *** $p < 0.001$; ** $p < 0.01$; * $p < 0.05$; ns, not significant.

Table S4. Two-way ANOVA results for willow plant data

Variable	df_A	F_A	*P_A*	Sig.	df_B	F_B	*P_B*	Sig.	df_AB	F_AB	*P_AB*	Sig.
Total biomass	1	626.303	*< 0.0001*	***	1	99.224	*< 0.0001*	***	1	18.843	0.0010	**
Root Pb	1	2.018	*0.1810*	ns	1	3.259	*0.0962*	ns	1	0.004	*0.9477*	ns
Stem Pb	1	2.140	*0.1692*	ns	1	0.092	*0.7664*	ns	1	0.492	*0.4964*	ns
Leaf Pb	1	3.078	*0.1048*	ns	1	0.003	*0.9595*	ns	1	0.387	*0.5458*	ns
Root Cu	1	6.235	*0.0281*	*	1	5.218	0.0414	*	1	6.156	*0.0289*	*
Stem Cu	1	0.166	*0.6906*	ns	1	6.723	0.0235	*	1	2.654	*0.1292*	ns
Leaf Cu	1	17.582	0.0012	**	1	59.646	*< 0.0001*	***	1	6.685	*0.0239*	*
Root Mn	1	5.534	0.0365	*	1	4.618	0.0527	ns	1	0.015	*0.9040*	ns
Stem Mn	1	7.740	*0.0166*	*	1	20.846	0.0006	***	1	4.902	*0.0469*	*
Leaf Mn	1	33.156	< 0.0001	***	1	43.892	< 0.0001	***	1	24.330	*0.0003*	***
Root Ni	1	5.023	0.0447	*	1	0.968	0.3446	ns	1	0.454	*0.5134*	ns
Stem Ni	1	1.330	*0.2713*	ns	1	0.777	*0.3955*	ns	1	0.165	*0.6914*	ns
Leaf Ni	1	5.247	0.0409	*	1	1.345	0.2687	ns	1	0.118	*0.7370*	ns
Root Zn	1	5.298	0.0401	*	1	1.876	0.1959	ns	1	8.514	*0.0129*	*
Stem Zn	1	11.149	0.0059	**	1	4.975	*0.0456*	*	1	1.505	*0.2435*	ns
Leaf Zn	1	1.775	*0.2075*	ns	1	0.412	*0.5331*	ns	1	1.728	*0.2132*	ns
Root Ca	1	3.842	0.0736	ns	1	0.718	0.4134	ns	1	7.554	*0.0177*	*
Stem Ca	1	0.154	*0.7013*	ns	1	0.008	*0.9300*	ns	1	1.923	0.1907	ns
Leaf Ca	1	6.013	*0.0305*	*	1	2.689	*0.1270*	ns	1	0.320	*0.5821*	ns
Root As	1	0.703	*0.4182*	ns	1	0.143	*0.7119*	ns	1	0.702	*0.4185*	ns
Stem As	1	6.172	*0.0287*	*	1	1.969	*0.1859*	ns	1	0.041	*0.8428*	ns
Leaf As	1	3.101	*0.1037*	ns	1	0.696	*0.4205*	ns	1	1.878	*0.1956*	ns

Note: Factor A = Planting mode (W vs WV-W); Factor B = 24-Epi treatment (Brs vs NBrs). *** $p < 0.001$; ** $p < 0.01$; * $p < 0.05$; ns, not significant.

Table S5. Two-way ANOVA results for vetiver plant data

Variable	df_A	F_A	*P_A*	Sig.	df_B	F_B	*P_B*	Sig.	df_AB	F_AB	*P_AB*	Sig.
Total biomass	1	14.628	0.0024	**	1	59.217	*< 0.0001*	***	1	1.506	*0.2433*	ns
Root Pb	1	0.067	*0.7998*	ns	1	2.506	*0.1394*	ns	1	3.765	*0.0762*	ns
Shoot Pb	1	0.579	*0.4614*	ns	1	0.014	*0.9085*	ns	1	2.089	*0.1740*	ns
Root Cu	1	0.708	*0.4164*	ns	1	0.204	*0.6596*	ns	1	1.093	*0.3163*	ns
Shoot Cu	1	1.209	*0.2931*	ns	1	30.862	*0.0001*	***	1	0.177	*0.6812*	ns
Root Mn	1	0.037	*0.8515*	ns	1	0.156	0.6998	ns	1	0.269	*0.6135*	ns
Shoot Mn	1	4.045	*0.0673*	ns	1	0.052	*0.8229*	ns	1	0.445	*0.5175*	ns
Root Ni	1	94.635	*< 0.0001*	***	1	1.806	0.2038	ns	1	2.267	*0.1580*	ns
Shoot Ni	1	0.082	0.7795	ns	1	85.440	*< 0.0001*	***	1	4.993	*0.0452*	*
Root Zn	1	3.112	*0.1031*	ns	1	0.105	*0.7510*	ns	1	0.456	*0.5123*	ns
Shoot Zn	1	0.033	*0.8594*	ns	1	10.007	*0.0082*	**	1	0.000	*0.9902*	ns
Root Ca	1	0.090	*0.7695*	ns	1	8.815	*0.0117*	*	1	0.270	*0.6129*	ns
Shoot Ca	1	0.014	*0.9065*	ns	1	0.000	*0.9953*	ns	1	1.015	*0.3337*	ns
Root As	1	0.155	*0.7012*	ns	1	0.407	*0.5354*	ns	1	0.206	*0.6580*	ns
Shoot As	1	6.110	*0.0294*	*	1	0.128	*0.7263*	ns	1	1.757	*0.2096*	ns

Note: Factor A = Planting mode (V vs WV-V); Factor B = 24-Epi treatment (Brs vs NBrs). *** $p < 0.001$; ** $p < 0.01$; * $p < 0.05$; ns, not significant.

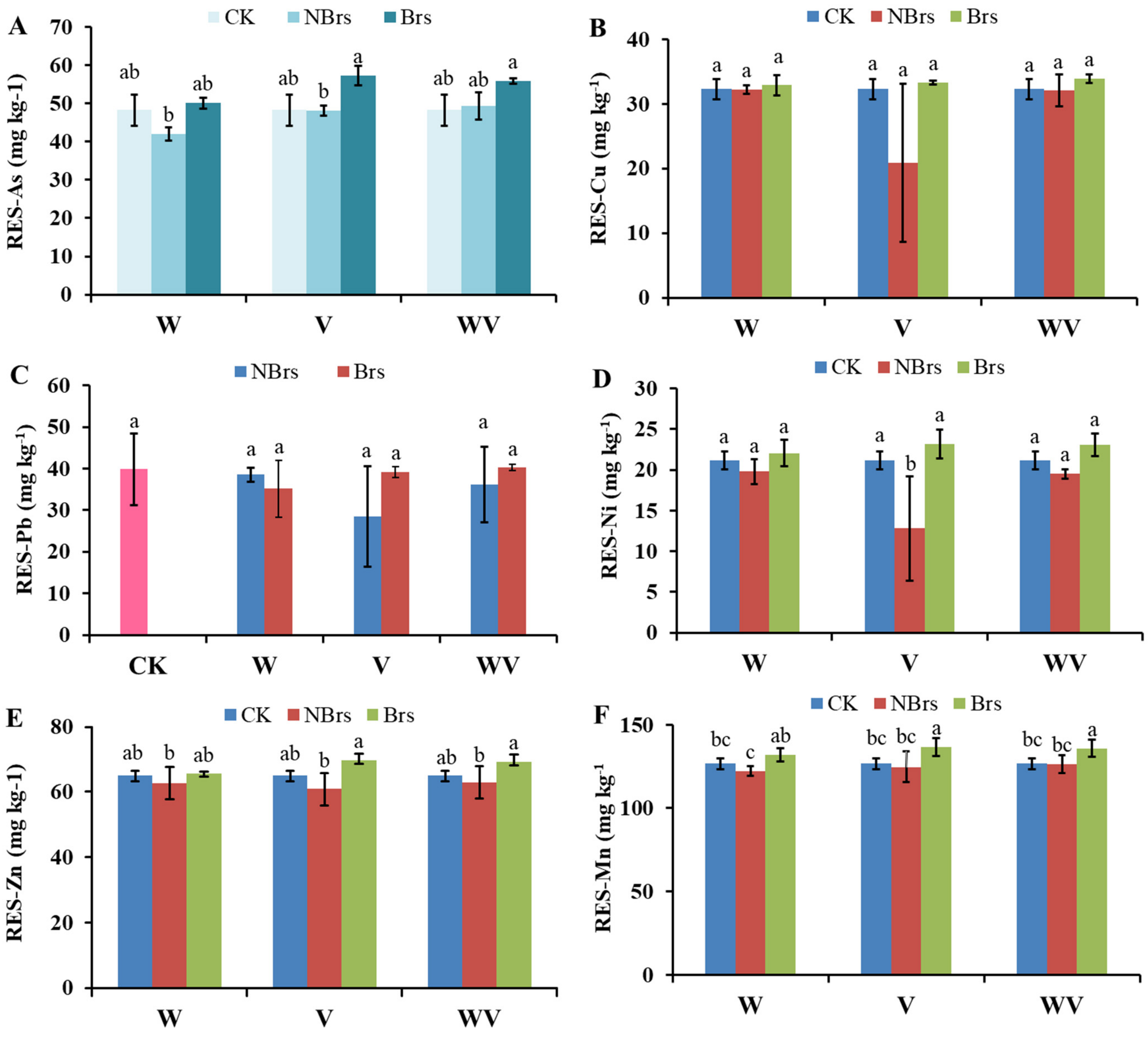

Figure S1 The residual fractions of soil As, Cu, Pb, Ni, Zn, and Mn
